# Genetic insights into antimicrobial resistance and virulence characteristics of *Salmonella enterica* isolated from Nile tilapia sourced from retail markets in Thailand

**DOI:** 10.1186/s12866-025-04451-0

**Published:** 2025-11-25

**Authors:** Justice Opare Odoi, Saran Anuntawirun, Nawaphorn Roongrojmongkhon, Worapong Singchat, Thitipong Panthum, Thomas Michael Roberts, Taane G. Clark, Kornsorn Srikulnath, Saharuetai Jeamsripong

**Affiliations:** 1 Department of Veterinary Public Health, Faculty of Veterinary Science, Chulalongkorn University, Bangkok, Thailand; 2 Animal Health Division, Animal Research Institute, Council for Scientific and Industrial Research, Accra, Ghana; 3 Animal Genomics and Bioresource Research Unit (AGB Research Unit), Faculty of Science, Kasetsart University, 50 Ngamwongwan, Chatuchak, Bangkok, 10900 Thailand; 4 Department of Infection Biology, Faculty of Infectious and Tropical Diseases,, London School of Hygiene and Tropical Medicine, Keppel Street, London, UK; 5 Faculty of Epidemiology and Population Health, London School of Hygiene and Tropical Medicine, Keppel Street, London, UK; 6 Department of Veterinary Public Health, Faculty of Veterinary Science, Research Unit in Microbial Food Safety and Antimicrobial Resistance, Chulalongkorn University, Bangkok, Thailand

**Keywords:** Antimicrobial resistance, Nile tilapia, *Salmonella enterica*, Virulence factors

## Abstract

**Background:**

Antimicrobial-resistant *Salmonella enterica* in aquaculture represents a significant public health concern due to its potential transmission through the food chain. Despite these concerns, there is a notable lack of comprehensive genomic characterization of *S. enterica* isolates from Nile tilapia in Thailand. This study aimed to evaluate the prevalence, antimicrobial resistance (AMR) profiles, and genetic diversity of *S. enterica* isolated from retail-sourced Nile tilapia (*Oreochromis niloticus*) between May and October 2023.

**Methods:**

A total of 714 Nile tilapia specimens comprising liver, kidney, meat, gill, mucus, lung, and intestine tissues were collected from fish purchased at retail markets in Thailand. *Salmonella* isolation and identification were performed using standard microbiological methods following ISO 6579-1:2017, and serotyping was conducted using the Kauffmann–White scheme. Antimicrobial susceptibility testing was carried out using the agar dilution method in accordance with CLSI guidelines. The whole genome sequencing was performed on 14 multidrug-resistant isolates using Illumina short-read technology to characterize their genetic resistance and virulence profiles.

**Results:**

The prevalence of *S. enterica* was 15.4%, with the highest rates observed in gill (40.9%) and mucus (26.5%) samples. Thirty-five serovars were identified, with Escanaba, Kentucky, and Othmarschen being the most frequently detected. Resistance to oxytetracycline (33.6%), tetracycline (16.0%), and ampicillin (12.8%) were prevalent, and 19.2% of isolates exhibited multidrug resistance (MDR). Although extended-spectrum beta-lactamase (ESBL)-producing isolates accounted for only 2.4%, concerns persist regarding their increasing global prevalence. WGS of 14 MDR isolates identified over 20 resistance genes, including *bla*_TEM−1B_, *bla*_CTX−M−55_, *qnrS1*, and *tet*(A), as well as efflux pump systems (*mdsABC* and *mdtK*) and various virulence factors.

**Conclusions:**

These isolates were classified into nine sequence types (STs), several of which have been associated with human infections. The findings underscore the necessity of stringent antimicrobial regulations and improved aquaculture practices to mitigate AMR transmission and enhance food safety.

**Supplementary Information:**

The online version contains supplementary material available at 10.1186/s12866-025-04451-0.

## Background

The increasing incidence of antimicrobial resistance (AMR) represents a pressing challenge in both human and veterinary medicine, with important implications for public health and environmental safety. Aquaculture has been identified as a major contributor to the proliferation of AMR bacteria [1–3], largely because of the extensive use of antimicrobials in fish farming. This practice inadvertently promotes the emergence and dissemination of resistant bacterial strains that can persist in aquatic environments and facilitate horizontal gene transfer. Additionally, aquatic environments, which are frequently contaminated by terrestrial effluents that originate from livestock production systems and sewage, serve as hotspots for AMR bacteria and resistance genes [4]. Comparative data indicates that livestock production and wastewater remain the dominant drivers of AMR in Southeast Asia, with multiple studies documenting a markedly higher prevalence of extended-spectrum beta-lactamase (ESBL)-producing *Enterobacterales* in poultry farms and municipal wastewater compared to the generally lower levels observed in aquaculture systems [5–7]. Nevertheless, the persistence and spread of AMR bacteria in aquaculture ecosystems not only undermine the efficacy of antimicrobial treatments, raise the risk of resistant pathogens entering the food chain through the consumption of contaminated fish and warrant the need for surveillance and sustainable management practices [8].

*Salmonella*, which is a bacterial genus recognized for its substantial impact on public health, has gained global prominence in the context of foodborne disease research. It remains a leading cause of foodborne diarrheal illness worldwide, with certain serotypes, such as *S.* Enteritidis and *S.* Typhimurium, capable of entering the bloodstream and causing septicemia [9]. In Southeast Asia, *Salmonella* has frequently been detected in various food products, which include chicken, pork, and fish [10–12]. According to the annual surveillance report of the Bureau of Epidemiology in Thailand, *Salmonella* is a major etiological agent of food poisoning, which often leads to hospitalization [13]. Consequently, its presence in food products poses substantial risks to consumers.

Nile tilapia (*Oreochromis niloticus*) is a key aquaculture species in Thailand, contributing significantly to national food security and rural income. Together, commercial tilapia and shrimp farming account for 0.38% of total GDP, generating value added of 96.2 and indirect economic impacts of approximately USD 35 million, along with the creation of around 16,000 additional jobs [14]. Thailand is among the world’s top tilapia producers, and its intensive farming practices, high stocking densities, and antimicrobial use create conditions that may favor the emergence and spread of AMR, making the region a critical setting for evaluating sustainability and health risks with insights applicable to other rapidly expanding tilapia industries in Asia and Africa. Nile tilapia has been increasingly recognized as a potential reservoir for *Salmonella* [15, 16], which raises critical food safety concerns. Recent studies have underscored the prevalence of AMR *Salmonella* in aquaculture environments and highlighted the urgency of this issue [16, 17]. For example, one study has reported a 45.5% prevalence of *Salmonella* in fresh tilapia samples and high resistance rates to multiple antibiotics, such as amoxicillin/clavulanic acid (87.7%), tetracycline (82.5%), and sulfonamides (57.9%). Key AMR genes, such as *bla*_CTX_ (beta-lactams), *tet*(B) (tetracycline), *sul2* (sulfonamide), and *floR* (chloramphenicol), have also been detected, which indicate a substantial presence of multidrug-resistant strains [18]. These findings emphasize the need for continuous monitoring and regulatory measures to ensure the safety of tilapia within the aquaculture supply chain.

Furthermore, virulence factors are crucial in determining the pathogenicity of *Salmonella* because they influence its ability to invade host tissues, evade immune responses, and induce disease [19]. The virulence of *Salmonella* is associated with diverse genetic elements, including virulence plasmids, pathogenicity islands, specific genes that encode toxins, and other virulence-associated proteins [19, 20]. Investigating the presence and distribution of these factors in Nile tilapia is essential for assessing potential health risks to consumers. Although previous studies have identified virulence factors in multidrug-resistant *Salmonella* isolates from different aquatic species [21, 22], comprehensive genomic data on isolates from Nile tilapia, particularly those that originate from Bangkok and the surrounding provinces, remain limited.

This study hypothesized that *S. enterica* isolated from farmed Nile tilapia would harbor resistance and virulence determinants that could contribute to both food safety concerns and public health risks. The aim of the study was to provide genomic insights into AMR and virulence factors in *Salmonella* isolated from Nile tilapia in Thailand by sequencing a subset of MDR using whole genome sequencing (WGS). These findings are anticipated to serve as a scientific basis for developing effective management and control strategies and contribute to global efforts to combat AMR and ensure food safety in aquaculture environments.

## Materials and methods

### Description of samples collected

**Fig. 1:**
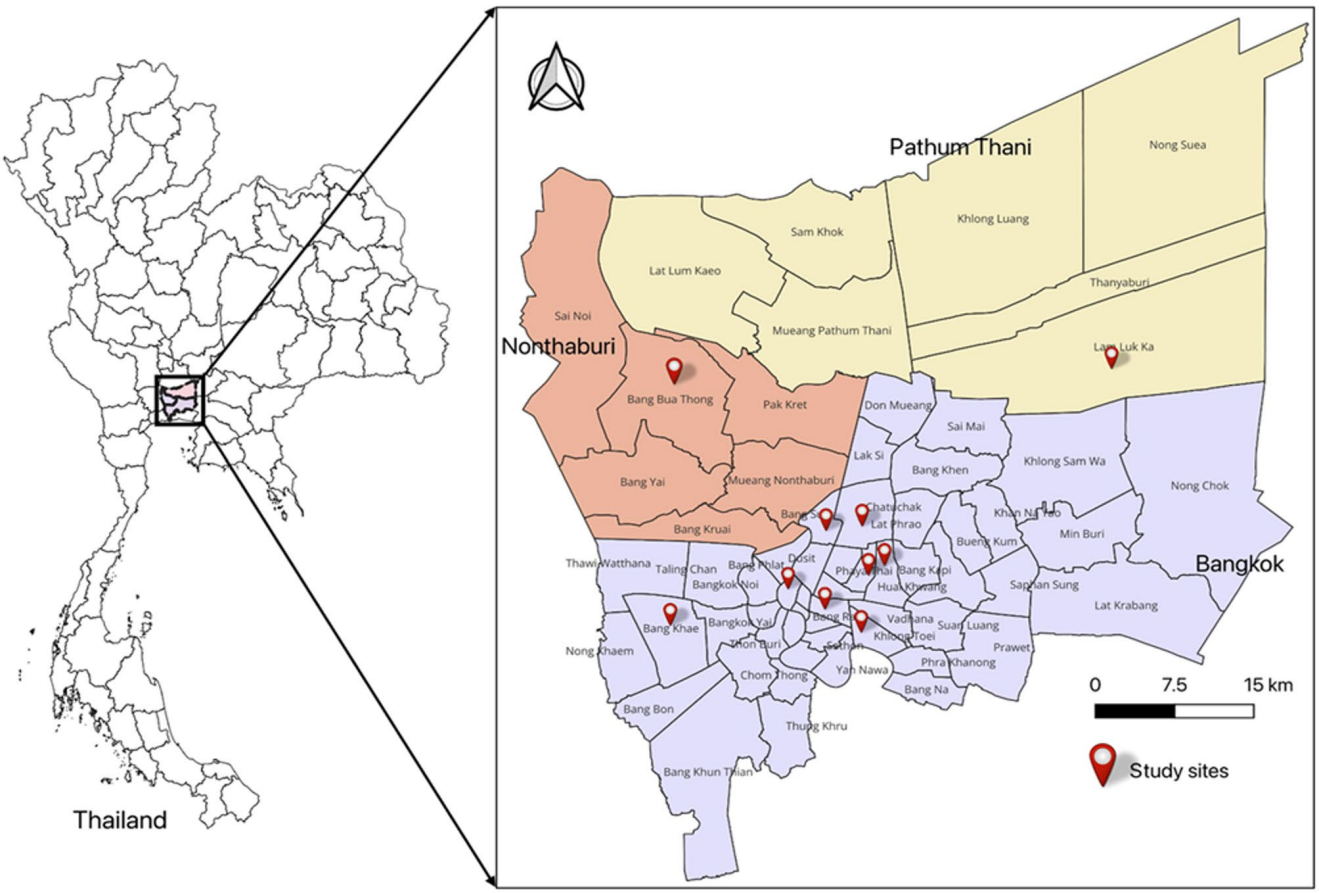
Distribution of sampling sites

Nile tilapia is the primary species farmed in Thailand and reaches a mature weight of 600–1,000 g within 90–150 days [23] Samples were collected from various markets and retail stores in Bangkok, Nonthaburi, and Pathum Thani between October 2022 and June 2023 (Table 1; Fig. 1). Fish samples were purchased, placed in individual Ziplock bags, and transported on ice to the Department of Veterinary Public Health at the Faculty of Veterinary Science, Chulalongkorn University, where microbiological analysis was performed.

**Table 1:** Distribution of sampling location

Province	District	Number of fish (%)	Number of specimens (%)
Bangkok	Khlong Toei	15 (10)	75 (10.5)
	Pathumwan	6 (4.0)	48 (6.7)
	Din Daeng	2 (1.3)	10 (1.4)
	Bang Sue	12 (8.0)	56 (7.8)
	Chatuchak	16 (10.7)	56 (7.8)
	Pom Prap Sattru Phai	40 (26.7)	196 (27.5)
	Huai Kwang	14 (9.3)	62 (8.7)
	Bang Kaen	6 (4.0)	20 (2.8)
Nonthaburi	Ngamwongwan	2 (1.3)	6 (0.8)
Pathum Thani	Lum Luk Ka	37 (24.7)	185 (25.9)
Total		150	714

To address ethical concerns related to tilapia sample collection, only dead fish sourced from markets were used. This approach negated the need for specific ethical approval for fish collection. Furthermore, the experimental use of pathogens in this study was reviewed and approved by the Faculty of Veterinary Science Biosafety Committee (CU-VET-BC) of Chulalongkorn University under reference number IBC 2,331,005.

### Sample processing

The weight (g), width (cm), and length (cm) of each fish were measured using a weighing scale (Sunford, Model FEH500, China) and measuring tape. The meat and internal organs, which included the gills, mucus, liver, kidney, and intestines, were carefully excised using a sterile surgical blade to facilitate bacterial isolation. A total of 714 tilapia specimens were sampled, comprising meat (*n* = 150), liver and kidney (*n* = 150), intestine (*n* = 150), mucus (*n* = 132), and gill (*n* = 132) samples.

### *Salmonella* isolation and identification

*Salmonella* isolation followed the ISO 6579-1:2017 standard. Meat (25 g), mucus (1 g), intestine (1 g), liver and kidney (1 g), and gills (1 g) were individually homogenized and mixed with lactose broth. All suspensions were incubated at 37 °C for 24 h, after which 100 µl aliquots were transferred to a modified semi-solid Rappaport–Vassiliadis (MSRV) (BD Difco, NJ, USA) medium and incubated at 42 °C for 24 h. To monitor potential cross-contamination during sampling and processing, uninoculated broth and agar media were included as negative controls and incubated under the same conditions as the inoculated samples; no growth was observed in these controls throughout the study.

To obtain distinct colony formations, a loopful of each suspension was streaked onto xylose lysine deoxycholate (XLD; BD Difco, NJ, USA), and MacConkey (BD Difco, NJ, USA) agar plates, followed by incubation at 37 °C for 24 h. Suspected *Salmonella* colonies appeared as pink or red colonies with or without black center spots on XLD, colorless colonies on MacConkey agar, and blue colonies with or without black center spots on Hektoen enteric agar plates.

To confirm *Salmonella* isolation, three suspected *Salmonella* colonies were biochemically confirmed on triple-sugar iron (TSI) (Difco) slant agar following the US Food and Drug Administration Bacteriological Analytical Manual method [24]. *Salmonella* colonies on the TSI slant after 24 h of incubation at 37 °C exhibited a red or purple slant and yellow butt with H_2_S production on TSI and tested positive for citrate utilization. Isolates confirmed as positive on TSI were stocked in Luria–Bertani broth that contained 20% glycerol and stored in a −80 °C freezer for further use.

### *Salmonella* serotyping

For serotyping, at least three *Salmonella* colonies were selected from each sample. A slide agglutination test was employed to determine the selected *Salmonella* serotype, and antigenic formulae were assigned using the Kauffmann–White scheme and Pasteur Institute classification [25]. Commercially available antisera (S&A Reagents Lab Ltd., Lat Phrao, Bangkok, Thailand) were used to examine the *Salmonella* serotype.

### Antimicrobial Susceptibility Testing (AST)

The agar dilution method, which followed the Clinical and Laboratory Standards Institute (CLSI) guidelines [26], was used to determine the minimum inhibitory concentrations (MICs) of various antimicrobials. The established breakpoints, in accordance with the CLSI guidelines [27] for 12 antimicrobials were as follows: ampicillin (AMP; 32 µg/mL), chloramphenicol (CHL; 32 µg/mL), ciprofloxacin (CIP; 4 µg/mL), enrofloxacin (ENR; 4 µg/mL), gentamicin (GEN; 8 µg/mL), ofloxacin (OXO; 8 µg/mL), oxytetracycline (OTC; 16 µg/mL), streptomycin (STR; 32 µg/mL), sulfamethoxazole (SUL; 512 µg/mL), tetracycline (TET; 16 µg/mL), trimethoprim (TRI; 16 µg/mL), and florfenicol (FLO; 8 µg/mL).

For quality control, the strains *Escherichia coli* ATCC 25,922, *Pseudomonas aeruginosa* ATCC 27,853, and *Staphylococcus aureus* ATCC 29,213 were used. Multidrug resistance (MDR) was defined as resistance to at least three different antimicrobial classes [28]. The multiple antibiotic resistance (MAR) index was calculated using a previously described formula [29].

$$\mathrm{MAR}\;\mathrm{index}\;=\;\frac{\mathrm{Number}\:\mathrm{of}\:\mathrm{antibiotics}\:\mathrm{to}\:\mathrm{which}\:\mathrm{the}\:\mathrm{isolate}\:\mathrm{is}\:\mathrm{resistant}}{\mathrm{Total}\:\mathrm{number}\:\mathrm{of}\:\mathrm{antibiotics}\:\mathrm{tested}}$$

### Detection of ESBL-producing *S. enterica*

The disk diffusion method, as described in the CLSI standard [30], was used to assess ESBL production. Ceftazidime (30 µg), cefotaxime (30 µg), and cefpodoxime (10 µg) disks were used for the initial screening of ESBL production in *S. enterica* isolates. Isolates resistant to at least one of these cephalosporins were subjected to further testing using a combination disk diffusion method with clavulanic acid. ESBL production was determined by measuring inhibition zones according to CLSI guidelines.

### Whole genome sequencing

WGS data, which consisted of Illumina paired-end short reads, were used to analyze the genetic background of the 14 microbial isolates. Quality control of the reads was conducted using FastQC version 0.12 [31]. Trimmomatic software (v 0.39) was used to remove adapters and crop the reads [32]. The filtered reads were then assembled using Spades (v 3.15) to construct contigs [33], and the quality of the output assembly was assessed using QUAST (v 5.2) [34].

The scaffolded genome assembly was used for antibiotic resistance and virulence gene detection using ABRicate (v1.0.1) with the ResFinder and VFDB databases, respectively (https://github.com/tseemann/abricate). Gene hierarchical clustering was performed using the hclust function from the core stats package in R (v 4.3.3). Multi-locus sequence typing (MLST) was performed using MLST Finder (https://cge.food.dtu.dk/services/MLST/), and sequences were compared against the PubMLST database to determine sequence types (STs).

Plasmid identification was performed by mapping the assembled contigs against the PlasmidFinder database (https://cge.cbs.dtu.dk/services/PlasmidFinder/) to detect plasmid-associated sequences. Mobile genetic elements (MGEs), including insertion sequences and transposons, were identified using the MobileElementFinder tool available at https://cge.food.dtu.dk/services/MobileElementFinder. A phylogenetic tree was constructed based on sequence-type data derived from WGS using the neighbor-joining method in MEGA 10 (available at https://www.megasoftware.net, accessed on August 20, 2024). Furthermore, to infer the evolutionary relationships among *Salmonella* isolates, a maximum likelihood phylogenetic tree using the 14 sequenced isolates from Nile tilapia was constructed alongside *Salmonella* sequences from diverse sources retrieved from GenBank to provide comparative context.

### Hierarchical clustering of AMR genes

A binary matrix, which represented the presence or absence of AMR genes in the 14 isolates, was constructed. Hierarchical clustering was performed using Euclidean distance metric and average linkage (UPGMA) method to analyze gene co-occurrence patterns. The analysis was conducted using the tools available on the Galaxy platform [35].

### Statistical analysis

Descriptive statistics were employed to assess the prevalence of resistance phenotypes and genotypes, resistance patterns, MDR, virulence genes, and ESBL production in *Salmonella* isolates. Chi-square tests were performed to evaluate differences in the rates of resistant *Salmonella* isolated from various sample types. A *p*-value of less than 0.05 was considered significant. A heatmap, which visually represented the distribution of resistant *Salmonella* across different sample types, was created using Displayr (http://www.displayr.com). All statistical analyses were performed using Stata version 14.0 (StataCorp, College Station, TX, USA).

## Results

### Distribution of collected samples

A total of 714 Nile tilapia specimens were collected from various locations in Bangkok, Nonthaburi, and Pathum Thani (Table 1; Fig. 1). The sampled fish had an average weight of 664.17 g (± 175.5 g), average width of 12.0 cm (± 1.2 cm), and average length of 30.2 cm (± 2.6 cm). Microbial analysis of the 714 Nile tilapia specimens revealed that 110 tested positive for *Salmonella*, which resulted in an overall prevalence of 15.4%. The samples consisted of mucus, gill, meat, liver and kidney, and intestine samples.

Significant differences (*p* < 0.05) in prevalence were observed across different sample types, with the highest positivity rate (40.9%) recorded in the gill samples, followed by mucus samples (26.5%). Lower prevalence rates were identified in the intestine and meat samples (7.3% and 5.3%, respectively), while the lowest positivity rate was observed in the liver and kidney samples (0.7%) (Table 2).

**Table 2:** Distribution of Salmonella stratified by sample type

Sample type	Sample quantity	Positive sample	% Positive
Mucus	132	35	26.5^b^
Gill	132	54	40.9^a^
Meat	150	9	5.3^c^
Liver & kidney	150	1	0.7^c^
Intestine	150	11	7.3^c^
Total	714	110	15.4

Values with different superscript letters are significantly different from each other (*p* < 0.05)

### Identified *Salmonella* serovars

A total of 35 *S. enterica* serovars were identified across the different fish specimens (Table 3). Among the three distinct sample types, the following serovars were detected: Agona, Braenderup, Escanaba, Senftenberg, Stanley, and Thies. Tallahassee and Othmarschen were detected in four different samples. Interestingly, except for the Kentucky serovar, no other serovars were detected in the liver and kidney samples.

**Table 3:** Distribution of *Salmonella* serovars in various tissues samples of Nile tilapia

Serovar	Sample type (%)					Total (*n* = 714)
	Mucus (*n* = 132)	Gill (*n* = 132)	Meat (*n* = 150)	Liver & kidney (*n* = 150)	Intestine (*n* = 150)	
Adjame	0 (0)	2 (1.5)	0 (0)	0 (0)	0 (0)	2 (0.3)
Agona	6 (4.6)	6 (4.5)	3 (2.0)	0 (0)	0 (0)	15 (2.1)
Amsterdam	3 (2.3)	0 (0)	0 (0)	0 (0)	0 (0)	3 (0.4)
Braenderup	9 (6.8)	3 (2.3)	3 (2.0)	0 (0)	0 (0)	15 (2.1)
Brandenberg	0 (0)	0 (0)	0 (0)	0 (0)	3 (2.0)	3 (0.4)
Brazil	2 (1.5)	0 (0)	0 (0)	0 (0)	0 (0)	2 (0.3)
Chiredzi	0 (0)	3 (2.3)	0 (0)	0 (0)	0 (0)	3 (0.4)
Cubana	0 (0)	12 (9.1)	0 (0)	0 (0)	0 (0)	12 (1.7)
Emek	1 (0.8)	0 (0)	0 (0)	0 (0)	0 (0)	1 (0.1)
Escanaba	12 (9.1)	42 (31.8)	6 (4.0)	0 (0)	0 (0)	60 (8.4)
Followfield	6 (4.6)	0 (0)	0 (0)	0 (0)	0 (0)	6 (0.8)
Georgia	0 (0)	0 (0)	3 (2.0)	0 (0)	0 (0)	3 (0.4)
Gombe	0 (0)	3 (2.3)	0 (0)	0 (0)	0 (0)	3 (0.4)
Isangi	3 (2.3)	1 (0.8)	0 (0)	0 (0)	0 (0)	4 (0.6)
Istanbul	0 (0)	0 (0)	0 (0)	0 (0)	2 (1.3)	2 (0.3)
Kentucky	0 (0)	0 (0)	0 (0)	3 (2.0)	0 (0)	3 (0.4)
Langeveld	1 (0.8)	3 (2.3)	0 (0)	0 (0)	0 (0)	4 (0.6)
Matopeni	0 (0)	4 (3.0)	0 (0)	0 (0)	1 (0.7)	5 (0.7)
Mbandaka	9 (6.8)	4 (3.0)	0 (0)	0 (0)	0 (0)	13 (1.8)
Mikawasima	0 (0)	6 (4.6)	0 (0)	0 (0)	0 (0)	6 (0.8)
Montevideo	0 (0)	3 (2.3)	0 (0)	0 (0)	0 (0)	3 (0.4)
Muenster	0 (0)	0 (0)	3 (2.0)	0 (0)	0 (0)	3 (0.4)
Newlands	3 (2.3)	0 (0)	0 (0)	0 (0)	0 (0)	3 (0.4)
Newport	0 (0)	3 (2.3)	0 (0)	0 (0)	0 (0)	3 (0.4)
Orion	0 (0)	1 (0.8)	0 (0)	0(0)	0 (0)	1 (0.1)
Othmarschen	19 (14.4)	11 (8.3)	3 (2.0)	0 (0)	4 (2.7)	37 (5.2)
Potsdam	0 (0)	6 (4.6)	0 (0)	0 (0)	0 (0)	6 (0.8)
Saintpul	0 (0)	0 (0)	0 (0)	0 (0)	3 (2.0)	3 (0.4)
Schwarzengrund	0 (0)	0 (0)	3 (2.0)	0 (0)	0 (0)	3 (0.4)
Senftenberg	2 (1.5)	4 (3.0)	0 (0)	0 (0)	2 (1.3)	8 (1.1)
Stanley	3 (2.3)	7 (5.3)	0 (0)	0 (0)	6 (4.0)	16 (2.2)
Stockholm	0 (0)	1 (0.8)	0 (0)	0 (0)	0 (0)	1 (0.1)
Tallahassee	20 (15.2)	26 (19.7)	3 (2.0)	0 (0)	3 (2.0)	52 (7.3)
Thies	6 (4.5)	0 (0)	3 (2.0)	0 (0)	3 (2.0)	12 (1.7)
Yarrabah	0 (0)	11 (8.3)	0 (0)	0 (0)	3 (2.0)	14 (2.0)

Variability in the prevalence of serovars was noted, with some exhibiting higher prevalence in particular tissues. For instance, the highest prevalence was recorded in the gill samples, in which Escanaba was detected most frequently (31.8%), followed by Tallahassee (19.7%). In the mucus samples, Othmarschen and Tallahassee were frequently detected (14.4% and 15.2%, respectively). Escanaba was the predominant serovar in meat samples (4.0%), whereas Stanley was most frequently detected in intestine samples (4.0%). Overall, Escanaba (60 isolates, 8.4%), Tallahassee (52 isolates, 7.3%), and Othmarschen (37 isolates, 5.2%) were the three most detected serovars.

### Resistance and susceptibility patterns in *S. enterica* isolates

**Fig. 2:**
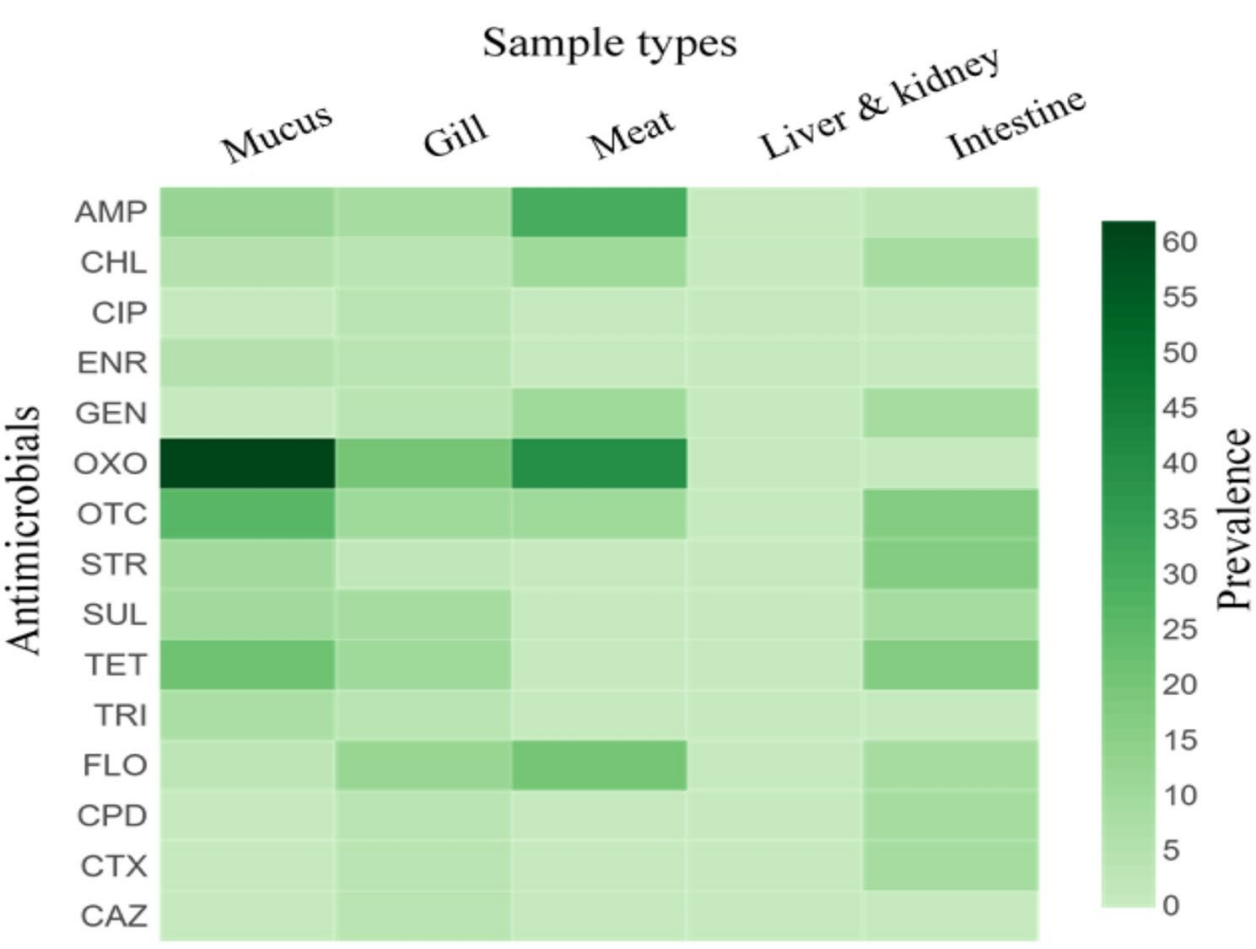
Distribution of *S. enterica* isolates resistant to various antimicrobials across different fish sample types. Note: AMP: ampicillin; CHL: chloramphenicol; CIP: ciprofloxacin; ENR: enrofloxacin; GEN: gentamicin; OXO: oxolinic acid; OTC: oxytetracycline; STR: streptomycin; SUL: sulfamethoxazole; TET: tetracycline; TRI: trimethoprim; FLO: florfenicol; CTX: cefotaxime; CAZ: ceftazidime; CPD: cefpodoxime

Among *S. enterica* isolates (*n* = 125), the highest resistance was observed against OXO (33.6%), followed by OTC (16.0%), TET (13.6%), and AMP (12.8%) (Table 4; Fig. 2). Notable differences in AMR profiles were identified across the different sample types in Nile tilapia. *S. enterica* isolates from the mucus samples showed resistance to ten of the antimicrobials investigated, which was predominantly against OXO (61.9%), followed by OTC (26.2%) and TET (21.4%). By contrast, resistance was observed against all 15 tested antimicrobials in the gill samples, with the highest resistance recorded for OXO (20.0%), FLO (11.7%), OTC (10.0%), and TET (10.0%). In the meat samples, the isolates showed resistance to six of the tested antimicrobials. The highest resistance was observed to OXO (40.0%), followed by AMP (30.0%) and FLO (20.0%). However, one isolate from the liver and kidney samples exhibited susceptibility to all tested antimicrobials. The isolates from the intestine samples demonstrated resistance to 10 of the tested antimicrobials, with the highest resistance observed for OTC, TET, and STR (16.7%). However, Pearson chi-square analysis indicated that no significant association was found between antimicrobial-resistant bacteria prevalence and sample type (*p* > 0.05).

**Table 4:** Antimicrobial susceptibility assessment for *S. enterica* (*n* = 125) isolated from Nile tilapia

Antimicrobials	Sample type (%)					Total (*n* = 125)
	Mucus (*n* = 42)	Gill (*n* = 60)	Meat (*n* = 10)	Liver & kidney (*n* = 1)	Intestine (*n* = 12)	
Ampicillin	5 (11.9)	5 (8.3)	3 (30.0)	0 (0)	3 (25.0)	16 (12.8)
Chloramphenicol	2 (4.8**)**	2 (3.3)	1 (10.0)	0 (0)	1 (8.3)	6 (4.8)
Ciprofloxacin	0 (0)	2 (3.3)	0 (0)	0 (0)	0 (0)	2 (1.6)
Enrofloxacin	2 (4.8)	2 (3.3)	0 (0)	0 (0)	0 (0)	4 (3.2)
Gentamicin	0 (0)	2 (3.3)	1 (10.0)	0 (0)	1 (8.3)	4 (3.2)
Oxolinic acid	26 (61.9)	12 (20.0)	4 (40.0)	0 (0)	0 (0)	42 (33.6)
Oxytetracycline	11 (26.2)	6 (10.0)	1 (10.0)	0 (0)	2 (16.7)	20 (16.0)
Streptomycin	4 (9.5)	1 (1.7)	0 (0)	0 (0)	2 (16.7)	7 (5.6)
Sulfamethoxazole	4 (9.5)	5 (8.33)	0 (0)	0 (0)	1 (8.3)	10 (8.0)
Tetracycline	9 (21.4)	6 (10.0)	0 (0)	0 (0)	2 (16.7)	17 (13.6)
Trimethoprim	3 (7.1)	2 (3.3)	0 (0)	0 (0)	0 (0)	5 (4.0)
Florfenicol	1 (2.4)	7 (11.7)	2 (20.0)	0 (0)	1 (8.3)	11 (8.8)
Cefpodoxime	0 (0)	2 (3.3)	0 (0)	0 (0)	1 (8.3)	3 (2.4)
Cefotaxime	0 (0)	2 (3.3)	0 (0)	0 (0)	1 (8.3)	3 (2.4)
Ceftazidime	0 (0)	2 (3.3)	0 (0)	0 (0)	0 (0)	2 (1.6)

### ESBL and MDR *Salmonella* isolate

Approximately 18.4% of the *Salmonella* isolates exhibited MDR, 28.8% demonstrated resistance to one or two antimicrobials, and 52.8% remained susceptible (Table 5). Among the 125 isolates, 7.2% exhibited resistance to four antimicrobial groups, 5.6% to three groups, 2.4% to five groups, 2.4% to nine groups, and 0.8% to both eight and seven groups. The MIC and zone of inhibition of the selected MDR are presented in Additional File Table S1. The detection rate of ESBL production in *S. enterica* isolates across various sample types was 2.4%. Four isolates from the gills (two isolates, 1.6%) and intestine (one isolate, 0.8%) were ESBL producers. The MAR indices for *S. enterica* and isolates recovered from the mucus, gill, meat, liver and kidney, and intestine samples were 0.11, 0.06, 0.08, 0 and 0.08, respectively (Table 6).

**Table 5:** Resistance profile of *S. enterica* (*n* = 125) isolated from Nile tilapia

AMR Pattern	Number of isolates from sample types													Total (%)	
	Mucus			Gills				Meat			Liver & kidney		Intestine		
Susceptible	13			39				4			1		9	66 (52.8)	
AMP-CHL-GEN-OTC-SUL-TET-FLO-CPD-CTX	0 (0)			0 (0)				0 (0)			0 (0)		1	1 (0.8)	
AMP-CHL-GEN-OXO-SUL-TRI-CPD-CTX-CAZ	0 (0)			2				0 (0)			0 (0)		0 (0)	2 (1.6)	
AMP-CHL-OXO-FLO	0 (0)			0 (0)				1			0 (0)		0 (0)	1 (0.8)	
AMP-CHL-OXO-OTC-STR-SUL-TET-TRI	1			0 (0)				0 (0)			0 (0)		0 (0)	1 (0.8)	
AMP-CIP-ENR-OXO	0 (0)			1				0 (0)			0 (0)		0 (0)	1 (0.8)	
AMP-ENR-OXO	0 (0)			1				0 (0)			0 (0)		0 (0)	1 (0.8)	
AMP-GEN	0 (0)			0 (0)				1			0 (0)		0 (0)	1 (0.8)	
AMP-OTC	1			0 (0)				0 (0)			0 (0)		0 (0)	1 (0.8)	
AMP-OTC-STR-TET	0 (0)			0 (0)				0 (0)			0 (0)		1	1 (0.8)	
AMP-OXO	1			0 (0)				0 (0)			0 (0)		0 (0)	1 (0.8)	
AMP-OXO-OTC	0 (0)			0 (0)				1			0 (0)		0 (0)	1 (0.8)	
AMP-OXO-OTC-STR-SUL-TET-TRI	1			0 (0)				0 (0)			0 (0)		0 (0)	1 (0.8)	
AMP-OXO-OTC-SUL-TET	0 (0)			1				0 (0)			0 (0)		0 (0)	1 (0.8)	
AMP-OXO-OTC-TET	1			0 (0)				0 (0)			0 (0)		0 (0)	1 (0.8)	
AMP-STR	0 (0)			0 (0)				0 (0)			0 (0)		1	1(0.8)	
CHL-OXO-STR-SUL-TRI	1			0 (0)				0 (0)			0 (0)		0 (0)	1 (0.8)	
ENR-OXO-OCT-TET	2			0 (0)				0 (0)			0 (0)		0 (0)	2 (1.6)	
FLO	1			7				1			0 (0)		0 (0)	9 (7.2)	
OTC	1			0 (0)				0 (0)			0 (0)		0 (0)	1 (0.8)	
OTC-STR-SUL-TET	0 (0)			1				0 (0)			0 (0)		0 (0)	1 (0.8)	
OTC-TET	0 (0)			1				0 (0)			0 (0)		0 (0)	1 (0.8)	
OXO	15			4				2			0 (0)		0 (0)	21 (16.8)	
OXO-OTC-SUL-TET	0 (0)			1				0 (0)			0 (0)		0 (0)	1 (0.8)	
OXO-OTC-TET	3			2				0 (0)			0 (0)		0 (0)	5 (4.8)	
OXO-OTC-STR-SUL-TET	1			0 (0)				0 (0)			0 (0)		0 (0)	1 (0.8)	

*AMP *ampicillin, *CHL *chloramphenicol, *CIP *ciprofloxacin, *ENR *enrofloxacin, *GEN *gentamicin, *OXO *oxolinic acid, *OTC *oxytetracycline, *STR *streptomycin, *SUL *sulfamethoxazole, *TET *tetracycline, *TRI *trimethoprim, *FLO *florfenicol, *CTX* cefotaxime, *CAZ *ceftazidime, *CPD *cefpodoxime

**Table 6:** Multiple antibiotic resistance (MAR) index of *S. enterica* across different Nile tilapia sample types

Sample type	No. of resistant isolates	MAR Index
Mucus	29	0.11
Gills	21	0.06
Meat	6	0.08
Liver & kidney	0	0
Intestine	3	0.08

### Sequence description

Assembly metrics for the 14 selected MDR isolates, which comprise the number of contigs, total genome length, GC content, total coding sequences, and N50, N90, L50, and L90 values, are presented (Additional file Table S2). The total genome length varied between 4.87 million (isolate M51.1) and 5.67 million (isolate I25.1) base pairs. The GC content remained consistent across isolates and ranged from 51.5% to 52.7%. The average number of coding sequences recorded was 5602. The N50 values ranged from 22,750 to 470,547 base pairs, whereas the N90 values ranged from 2,578 to 108,954 base pairs. The L50 values ranged from 4 to 75, while the L90 values ranged from 12 to 367 across the isolates.

### Genetic description of sequenced *Salmonella* isolates

WGS of the 14 isolates revealed that they belonged to nine ST lineages: ST13 (M75.1, MU39.1), ST26 (M51.1), ST34 (G26.3, G28.1, MU25.1), ST321 (MU84.1), ST413 (G76.3, MU78.1), ST446 (MU23.1), ST469 (I19.3), ST1541 (G71.1, G75.1), and ST2390 (I25.1). The maximum likelihood phylogeny revealed that *S. enterica* isolates from Nile tilapia in Thailand were distributed across several serovars, including *S.* Agona, *S.* Mbandaka, *S.* Stanley, *S.* Newlands, and *S.* Senftenberg (Additional file Fig. S1). These isolates clustered with reference genomes of the same serovars from diverse sources and regions, including humans, poultry, farms, food, and wet markets, indicating broad genetic diversity across hosts. The clustering pattern suggests that tilapia-associated isolates do not represent a single clonal population but rather reflect multiple introductions, potentially from environmental, agricultural, or food-chain sources.

**Fig. 3:**
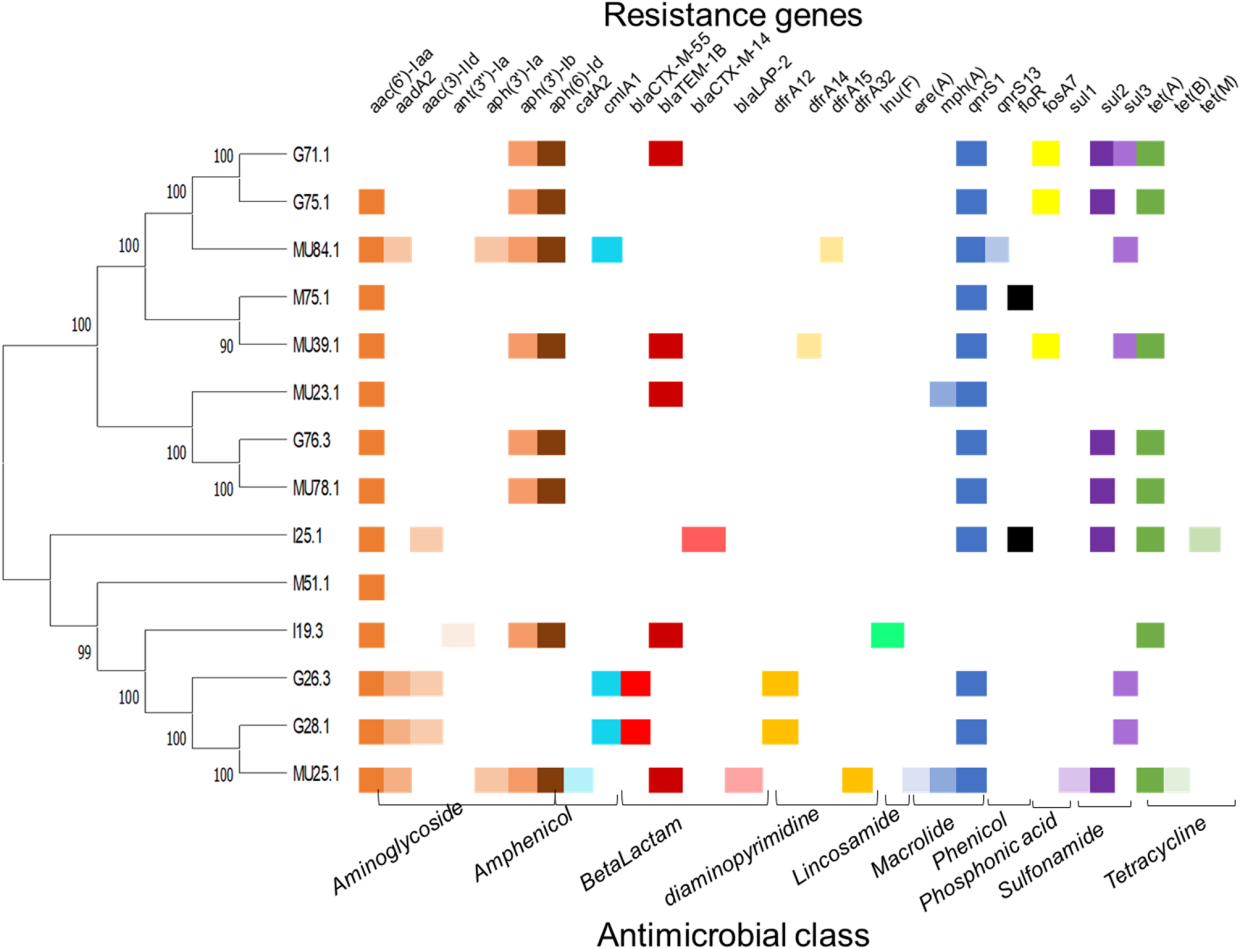
Phylogenetic representation and the presence of resistance genes in multidrug-resistant (MDR) isolates. In the isolate IDs, the letter indicates the sample source (G = gill, I = intestine, M = meat, MU = mucus), and the number represents the strain. The identified resistance genes are displayed above the chart, while the classes of antimicrobial agents to which resistance was detected are listed below the chart. Different colors in the chart represent various antibiotic classes associated with identified resistance genes: shades of brown indicate aminoglycosides, shades of turquoise represent amphenicol, shades of red signify beta-lactams, orange denotes diaminopyrimidines, bright green represents lincosamides, shades of blue indicate macrolides, black represents phenicols, yellow indicates phosphonic acids, shades of mauve represent sulfonamides, and shades of dull green signify tetracycline

The WGS data also revealed several resistance genes, which contributed to the MDR observed across all isolates (Fig. 3; Additional file Table S3). Over 20 different resistance genes were identified, and resistance was conferred to various antibiotic classes, including aminoglycosides (*aac*(6’)-Iaa, *aad*A2, *aph*(3’’)-Ib, *aph*(6)-Id), amphenicols (*cat*A2, cmlA1), beta-lactams (*bla*_TEM−1B_, *bla*_CTX−M−55_, *bla*_CTX−M−14_, *bla*_LAP−2_), diaminopyrimidines (*dfr*A12, *dfr*A14, *dfr*A15, *dfr*A32), lincosamides (*lnu*F), macrolides (*ere*(A), *mph*(A)), fluoroquinolones (*qnr*S1, *qnr*S13), phenicols (*flo*R), phosphonic acids (*fos*A7), sulfonamides (*sul*1, *sul*2, *sul*3), and tetracyclines (*tet*(A), *tet*(B), *tet*(M)). In addition, genes that encoded efflux pump systems, such as the *mds*ABC complex and *mdt*K, were detected in all isolates except for I25.1, which contained only the *mdt*K gene.

### Mobile Genetic Elements (MGEs) and their association with AMR genes

Further analysis of *S. enterica* isolates revealed the widespread presence of MGEs, which included miniature inverted repeats (MITEEc1) and various insertion sequences (IS) (Additional file Table S4). A total of 21 unique MGEs were identified across the 14 isolates.

Several MGEs were associated with resistance genes: ISEc9 in isolates G26.3 and G28.1 was linked to *bla*_CTX−M−55_ and *qnr*S1, whereas IS26 in isolates G71.1, G75.1 and G76.3 was associated with *aph*(6)-Id, *tet*(A), *aph*(3”)-Ib, and *sul*2. ISVsa3 and ISEc9 in isolate I25.1, were linked to *sul2* and *floR* and *qnr*S1, *bla*_CTX−M−55_, respectively. In addition, IS6100 and ISKpn19 in isolate MU23.1 were connected to *bla*_TEM−1B_, *tet*(A), *mph*(A), and *qnr*S1. In isolate MU25.1, ISKpn19 was located near *bla*_LAP−2_, *qnr*S1, and *mph*(A), whereas in isolate MU39.1, ISKpn19 was associated with *tet*(A) and *qnr*S1. IS903 in MU39.1 was linked to *aph*(3”)-Ib and *aph*(6)-Id. In isolate MU78.1, IS26 was found alongside *aph*(3”)-Ib, *aph*(6)-Id, *qnr*S1, *sul*2, and *tet*(A). Similarly, in MU84.1, the genetic environment of *aph*(3”)-Ib and *aph*(6)-Id included ISKpn18.

### Virulence factors and genes

**Fig. 4:**
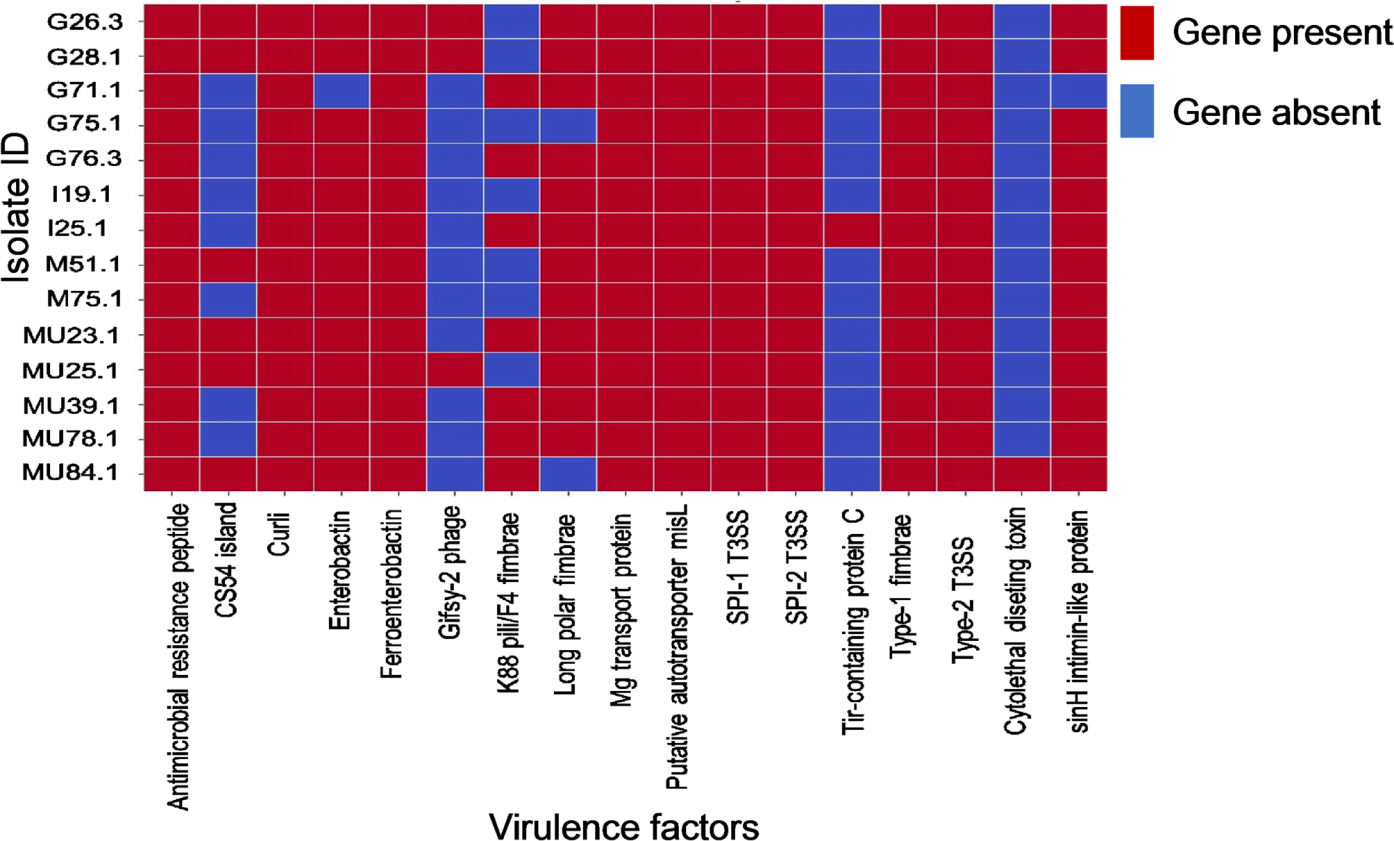
Variability in virulence factor distribution in *Salmonella* isolates. Red boxes show the presence of genes that encode virulence factors, whereas the blue colors indicate the absence of virulence factors

A total of 112 virulence genes associated with 17 virulence factors were identified in the *S. enterica* isolates. These factors included antimicrobial peptide resistance (*mig-14*), CS54 Island, curli, enterobactin, ferroenterobactin, *gifsy-2* phage, K88 pili/F4 fimbriae, long polar fimbriae, magnesium transport protein, putative autotransporter (*misL*), SPI-1 and SPI-2 type III secretion systems (T3SS), *tir*-containing protein C, type-1 fimbriae, type-2 T3SS, cytolethal distending toxin, and intimin-like protein (Fig. 4). Several virulence genes were present in all isolates. However, *cdtB* was detected exclusively in isolate MU84.1, whereas *tcpC* was only found in isolate I25.1. Notably, genes that encoded both Type 1 and Type 2 *Salmonella* pathogenicity islands (SPI-1 and SPI-2) were detected in all isolates, which highlighted their widespread presence across the analyzed strains (Fig. 4; Additional file Table S5).

### Clustering patterns of resistance genes

**Fig. 5:**
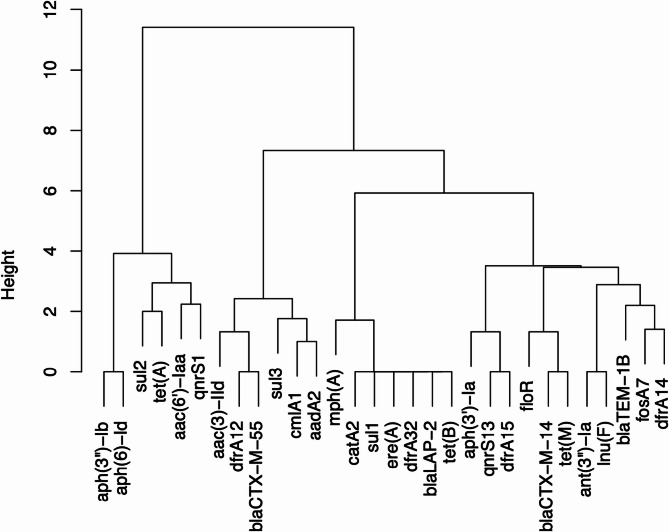
Hierarchical clustering of antimicrobial resistance genes among multidrug-resistant (MDR) *S. enterica* isolates. X-axis: Resistance genes; Y-axis: Clustering height. Genes that are clustered together are frequently co-detected within the same isolates

The dendrogram revealed multiple clusters of resistance genes that frequently co-occurred among *Salmonella* isolates. Certain genes, such as *sul2*, *tet*(A), and *aac(6’)-laa*, were found in close proximity and indicated potential co-selection or shared genomic elements. Similarly, *bla*_CTX−M−55_, *sul3*, and *dfrA12* formed a distinct cluster and indicated their possible association with MDR plasmids. The observed clustering patterns demonstrated the genetic complexity and potential mobility of the resistance determinants in aquaculture-related *Salmonella* strains (Fig. 5).

## Discussion

The overall prevalence of *S. enterica* in Nile tilapia observed in this study was 15.4%. This falls within the range of prevalence previously reported in Thailand, where rates have varied from as low as 7% to as high as 36% [16, 36]. Similarly, studies from other Southeast Asian countries have reported higher prevalence rates than those observed in our study, including 16% in Malaysia, 33% in Cambodia, and 41% in Vietnam [37–39]. These differences likely reflect variation in aquaculture practices, environmental conditions, and biosecurity measures, as well as differences in study scope and methodologies.

Importantly, because *S. enterica* is not a natural component of the normal fish microbiota, its detection in tilapia indicates potential environmental contamination or post-harvest contamination of the food chain. The higher prevalence detected in gill and mucus samples likely reflects their role as external tissues in direct contact with the aquatic environment, making them more prone to harbour waterborne bacteria rather than reflecting systemic infection. This assertion aligns with previous research showing that external tissues in fish can act as key ecological niches for microbial persistence [40, 41]. Longitudinal studies and routine surveillance are needed to track changes in *S. enterica* prevalence and identify contributing factors, while detection in retail fish addresses the importance of strict sanitation and regular microbial monitoring to protect consumers.

Identifying 35 *S. enterica* serovars across various fish specimens confirmed the diversity and distribution of these serovars (Table 3). This finding is consistent with that of similar research conducted in different geographical regions, which indicates a global pattern of *S. enterica* diversity in aquatic environments [42]. The presence of serovars, such as Escanaba in tilapia from Thailand, Tallahassee in aquatic environments in South Africa and Othmarschen from carcasses in Colombia and high prevalence rates observed in this study, highlight their widespread occurrence and increased potential for contamination [17, 43, 44]. These serovars pose an increased risk to human health if contaminated fish enter the food supply chain. In addition, the frequent detection of these serovars across multiple sample types suggests their adaptability and persistence in aquaculture environments. Interestingly, the absence of certain serovars in liver and kidney samples, except for Kentucky, suggests that specific serovars exhibit tissue tropism or a preference for host environments. Although some serovars appear capable of colonizing multiple tissues, others may be restricted to specific sites within the host. Further research is required to assess the tissue-specific prevalence of *Salmonella* serovars, which may help design targeted interventions for infection control.

The AST conducted on *S. enterica* isolates revealed that the majority showed resistance to OXO, OTC, TET, and AMP (Table 4). This finding is consistent with that of a study conducted to characterize AMR bacteria isolated from hybrid red tilapia in Thailand, in which *S. enterica* exhibited high resistance to AMP, OXO, and OTC [17]. The widespread use of OXO, OTC, and TET in aquaculture has resulted in numerous reports documenting bacterial resistance to these antimicrobials [8, 45], which may have contributed to the resistance observed in *S. enterica* isolates.

A limitation of this study was the inability to specifically determine the antimicrobial usage in aquatic farms. However, the relationship between antimicrobial consumption and resistance emergence is well established [46]. In contrast, resistance to CIP, CTX, CAZ, and CPD among *Salmonella* isolates was minimal. This is likely because of the limited use of these antimicrobials in aquaculture settings, which may have contributed to the low levels of resistance observed in these isolates. From a public health perspective, this situation is encouraging as it preserves the effectiveness of these antimicrobials for treating *Salmonella* infections.

The prevalence of ESBL-producing *S. enterica* in this study was 2.4%. This relatively low prevalence is noteworthy, given the global concern regarding the rising incidence of ESBL-producing bacteria, which exhibit resistance to a broad spectrum of beta-lactam antibiotics, including penicillins and cephalosporins [47]. Comparatively, a study conducted in South Africa reported a higher ESBL *Salmonella* prevalence (14.4%) in aquatic environments [43]. Another study conducted in Cambodia reported an even higher prevalence (17%) of ESBL-*Salmonella* infection in retail fish [48]. Although the ESBL prevalence observed in this study was not alarmingly high, it still warrants attention because ESBL-producing strains, even at a low prevalence, pose a risk for potential outbreaks and resistance gene dissemination.

The MAR index values for *S. enterica* isolated from various parts of the Nile tilapia (liver, kidney, meat, lungs, gills, mucus, and intestines) were lower than the minimum threshold of 0.2 (Table 6). The MAR index serves as a valuable tool for assessing the risk associated with the spread of AMR, with an index greater than 0.2 indicating extensive antimicrobial exposure in the environment from which the isolate originated [29]. In this study, the recorded MAR index values remained below 0.2 (Table 6), which suggested relatively low antimicrobial exposure in the aquaculture environment from which the fish were sourced. These findings have important implications as they indicate that current antimicrobial practices in the aquaculture environment studied may not substantially contribute to *S. enterica* resistance.

Although the study refers to the One Health framework, direct, data-driven connections between tilapia isolates and potential human, animal, or wastewater reservoirs were not established, and therefore transmission among these three domains cannot be addressed within the scope of this study. These results suggest that implementing stringent regulations on antimicrobial use in aquaculture and promoting good management practices can effectively control AMR spread. Consequently, continuous monitoring and periodic assessments of MAR index values in various aquaculture environments are essential for the early detection of any increase in resistance levels, which allows for the implementation of timely intervention measures.

In this study, 18.4% of the isolates were identified as MDR. Comparatively, *Salmonella* isolates from chickens (33%) and pigs (81%) have been reported to exhibit resistance to three or more antimicrobial classes [49], suggesting that the MDR burden in livestock systems is substantially higher than that observed in Nile tilapia, thereby reinforcing the need for sector-specific AMR mitigation strategies. The WGS analysis of the 14 *S. enterica* isolates from Nile tilapia provided important insights into their genetic backgrounds and potential mechanisms that contributed to MDR. However, future studies incorporating both MDR and non-MDR isolates will be important to better understand resistance dynamics in tilapia aquaculture. Nine STs of *S. enterica* were identified across different tissues of Nile tilapia, each associated with distinct AMR profiles (Additional file Table S3). The most frequently identified STs in this study were ST34, followed by ST13, ST413, and ST1541. Although ST34 was found in Nile tilapia in our study, it has not been previously reported in tilapia; however, it is commonly reported in food animals and human patients in Thailand [50, 51]. Though WGS provides important insights into the genetic basis of antimicrobial resistance and virulence in *Salmonella enterica* from aquaculture environments, it is not sufficient to predict public health risk in isolation; such assessments require integration with epidemiological data and clinical outcomes to establish transmission pathways and infection potential.ST34, which was detected in the gill and mucus samples (G26.3, G28.1, and MU25.1), carried multiple resistance genes, such as *bla*_CTX−M−55_. ST1541, which was detected in the gill samples (G71.1, G75.1), harbored *bla*_TEM−1B_. ST13, which was isolated from the meat and mucus samples (M75.1, MU39.1), contained *bla*_CTX−M−14_. ST413, which was isolated from the gill and mucus samples (G76.3, MU78.1), was associated with *qnrS1*. ST321, found in the mucus samples (MU84.1), carried *sul3*. ST26 (M51.1, meat) and ST446 (MU23.1, mucus) were linked to *aac(6’)-Iaa* and *bla*_TEM−1B_, respectively. ST469 (I19.3, intestine) was associated with *bla*_TEM−1B_, whereas ST2390 (I25.1, intestine) harbored *bla*_CTX−M−14_ and *qnrS1*.

This diversity in STs indicates a broad genetic variation among *S. enterica* populations associated with Nile tilapia. Such diversity may reflect the different ecological niches that these bacteria occupy within fish or the surrounding environment and their potential pathogenicity and antibiotic resistance profiles [52]. Additionally, several of the *S. enterica* sequence lineages, which included ST13, ST26, ST34, and ST413, have been implicated in human clinical infections and poultry-associated *Salmonella* contamination [21, 53, 54].

The detection of MDR genes, which include those conferring resistance to aminoglycosides, beta-lactams, and tetracyclines, underscores the potential impact of antibiotic use in aquaculture on the emergence and spread of resistant bacterial strains [55]. The presence of efflux pump systems, such as *mds*ABC complex and *mdt*K, aligns with the findings of previous studies on bacterial resistance mechanisms, in which efflux pumps have been identified as key contributors to MDR [56]. However, no association was observed between AMR phenotype and genotype, which may be attributable to the relatively small sample size of the genomic analysis.

Multiple MGEs, which include various insertion sequences (e.g., IS26, ISEc9, ISKpn19, IS6100, IS903, ISSen1, ISEcl10, and ISVsa3), a miniature inverted-repeat transposable element (MITEEc1), and a transposon (Tn6024) were detected across all *S. enterica* STs identified in this study (Additional file Table S4). The presence of multiple MGEs has been widely reported in *S. enterica* from clinical isolates [57]; however, *S. enterica* possessing single or no MGEs has also been documented [58]. Several MGEs were found to be associated with resistance determinants, such as *bla*_CTX−M−55_, *qnr*S1, and *aph*(3”)-Ib/*aph*(6)-Id. Notably, IS26 has frequently been implicated in resistance gene mobilization in aquatic environments [59]. No plasmids were detected in our isolates, indicating the resistance genes are not plasmid-borne. Thus, while MGEs may contribute to resistance dissemination, their functional role remains putative.

Moreover, several virulence genes were detected across all *S. enterica* STs. However, certain virulence genes were uniquely identified in specific STs, which suggested variable virulence potentials among the isolates. For instance, the *cdtB* gene, which encodes a subunit of the cytolethal distending toxin associated with DNA damage and cell cycle arrest, was detected exclusively in isolate MU84.1 (ST321). The *tcpC* gene, known to modulate host immune responses by interfering with Toll-like receptor signaling, was uniquely identified in isolate I25.1 (ST2390). Additionally, the *shdA* gene, which contributed to intestinal persistence and adhesion, was observed in isolates M51.1 (ST26) and MU23.1 (ST446), whereas *sodCl*, which encoded a periplasmic superoxide dismutase that enhanced bacterial survival within macrophages, was identified in isolates G26.3, G28.1, and MU25.1, all of which belong to ST34.

These findings suggest that while certain virulence determinants are broadly distributed, others may be lineage specific. This reflects the pathogenic diversity among the isolates [60, 61]. The universal detection of SPI-1 and SPI-2 across all isolates point to their role as core virulence determination of *Salmonella* and is consistent with a previous study [62]. However, the presence of SPI-1 and SPI-2 alone does not necessarily indicate heightened pathogenicity.

The phylogenetic clustering of tilapia isolates with strains from humans, poultry, and food highlights the interrelatedness of *Salmonella* under the One Health framework (Additional file Fig. S1). This observation suggests possible spillover events between aquaculture systems and other reservoirs, though our findings alone cannot establish direct transmission. The absence of plasmid-borne AMR genes in the sequenced isolates, combined with the relatively small sample size, underscores the need for cautious interpretation. Future studies employing larger datasets, longitudinal sampling, and metagenomic approaches will be critical to assess the persistence, transmission dynamics, and more precisely define the contribution of aquaculture to the broader AMR burden.

## Conclusions

Detecting MDR- and ESBL-producing *S. enterica* in Nile tilapia from aquaculture systems underscores severe threats to food safety and public health. The presence of diverse resistance genes and STs, some of which have been linked to human infections, suggests that aquaculture environments may act as reservoirs and transmission pathways for AMR bacteria. In addition, the identification of multiple virulence factors, which include those associated with adhesion, immune evasion, and toxin production, raises additional concerns about the pathogenic potential of these isolates, such traits can enhance bacterial persistence and facilitate host infection. The absence of plasmid-borne resistance genes, the relatively low prevalence of ESBL-producing isolates, and antimicrobial selection pressure in the studied aquaculture setting is limited. Thus, while tilapia-associated *S. enterica* harbors resistance and virulence determinants of potential concern, their broader ecological and clinical significance remains to be fully established. This study contributes valuable genomic data, but future research integrating longitudinal surveillance with genomic comparisons will be needed to better define the role of aquaculture in AMR dissemination. Strengthening stewardship practices and implementing continuous monitoring will be essential for safeguarding public health while supporting the long-term sustainability of aquaculture.

## Supplementary Information

Addtional file 1: Table S1. Minimum inhibitory concentration, zone of inhibition and resistance gene profile of multidrug resistant isolates. This file provides minimum inhibitory concentration and genotypic profile of the 14 whole genome sequenced isolates

Additional file 2: Table S2. Comparative sequence data for isolates Description of data: This file contains the comparative sequence data for all*Salmonella enterica* isolates analyzed in the study.

Additional file 3: Table S3. Antibiogram and detailed genomic profile of *Salmonella* isolates from Nile Tilapia.Description of data: This file provides the antimicrobial susceptibility profiles and detailed genomic characteristics of *Salmonella enterica* isolates obtained from Nile Tilapia.

Additional file 4: Table S4. Identified mobile genetic elements and their association with resistance genesDescription of data: This file presents a list of mobile genetic elements detected in *S. enterica* isolates and their associated AMR genes.

Additional file 5: Table S5. Distribution variability of virulence genes across *Salmonella enterica* isolates.Description of data: This file shows the variability in virulence gene profiles across all *S. enterica* isolates examined in the study.

Addtional file 6: Figure S1. Maximum likelihood phylogenetic tree of *Salmonella enterica* isolates from Nile tilapia in Thailand and reference genomes from GenBank. A phylogenetic tree was constructed using the maximum likelihood method based on core genome alignments of *Salmonella enterica* isolates sequenced in this study (n = 14), along with representative reference genomes obtained from GenBank. Isolates are labeled by genus and serovar, followed by accession numbers for GenBank references. Study isolates are denoted by alphabetical codes with numerical identifiers. Colors indicate the source of isolation, including fish tissues (gill, mucus, intestine, meat), humans, poultry, wastewater, food, farms, and wet markets. Country labels denote the geographical origin of each isolate. The scale bar (0.01) represents the number of nucleotide substitutions per site.

## Data Availability

The WGS data has been submitted to NCBI GenBank under the BioProject ID PRJNA1159476 with various accession number (JBHHKO000000000, JBHHKP000000000, JBHHKQ000000000, JBMPID000000000 JBHHKR000000000, JBHHKS000000000, JBHHKT000000000, JBHHKU000000000, JBHHKV000000000, JBHHKW000000000, JBHHKX000000000, JBHHKY000000000, JBHHKZ000000000, and JBHHLA000000000).

## Abbreviations

AMP: Ampicillin
AMR: Antimicrobial resistance
AST: Antimicrobial susceptibility testing
CAZ: Ceftazidime
CHL: Chloramphenicol
CIP: Ciprofloxacin
CLSI: Clinical and Laboratory Standards Institute
CPD: Cefpodoxime
CTX: Cefotaxime
ENR: Enrofloxacin
ESBL: Extended spectrum beta-lactamase
FLO: Florfenicol
GEN: Gentamicin
MAR: Multiple antibiotic resistance
MDR: Multidrug resistance
MGE: Mobile genetic elements
MSRV: Modified semi-solid Rappaport–Vassiliadis
OTC: Oxytetracycline
OXO: Oxolinic acid
STR: Streptomycin
SUL: Sulfamethoxazole
TET: tetracycline
TRI: Trimethoprim
TST: Triple sugar iron
WGS: Whole genome sequencing
XLD: Xylose lysine deoxycholate
