# Supplementary material for: Genetic insights into antimicrobial resistance and virulence characteristics of Salmonella enterica isolated from Nile tilapia sourced from retail markets in Thailand
Source: BMC Microbiol. 2025 Nov 25;25:777. doi: 10.1186/s12866-025-04451-0 (PMC12649085; doi:10.1186/s12866-025-04451-0)
Supplement: Supplementary file 2 — Additional file 2: Table S2. Comparative sequence data for isolates Description of data: This file contains the comparative sequence data for allSalmonella enterica isolates analyzed in the study. [file 12866_2025_4451_MOESM2_ESM.docx]

**Additional file**

**Table S2**. Comparative sequence data for isolates

| **Isolates** | **Accession Number** | **No. contigs** | **Total length** | **GC (%)** | **No. coding sequence** | **N50** | **N90** | **L50** | **L90** |
| --- | --- | --- | --- | --- | --- | --- | --- | --- | --- |
| G26.3 | JBHHKO000000000 | 305 | 5,266,194 | 51.5 | 5591 | 222750 | 43539 | 9 | 29 |
| G28.1 | JBHHKP000000000 | 311 | 5,268,590 | 51.9 | 5612 | 222750 | 43423 | 9 | 29 |
| G71.1 | JBMPID000000000 | 227 | 5,015,860 | 52.0 | 5256 | 410784 | 108014 | 5 | 13 |
| G75.1 | JBHHKQ000000000 | 228 | 5,015,868 | 52.1 | 5256 | 410784 | 108014 | 5 | 13 |
| G76.3 | JBHHKR000000000 | 337 | 5,081,205 | 52 | 5388 | 276786 | 80290 | 5 | 19 |
| I19.3 | JBHHKS000000000 | 543 | 5,421,317 | 51.8 | 5868 | 115900 | 14664 | 16 | 54 |
| I25.1 | JBHHKT000000000 | 721 | 5,666,655 | 51.7 | 6356 | 112713 | 18187 | 16 | 57 |
| M51.1 | JBHHKU000000000 | 268 | 4,865,686 | 52.3 | 5040 | 463540 | 108954 | 4 | 12 |
| M75.1 | JBHHKV000000000 | 1078 | 5,152,747 | 52.7 | 6055 | 307935 | 88736 | 5 | 17 |
| MU23.1 | JBHHKW000000000 | 371 | 4,977,676 | 52.1 | 5214 | 470547 | 91169 | 4 | 13 |
| MU25.1 | JBHHKX000000000 | 403 | 5,313,971 | 52 | 5703 | 262579 | 41206 | 8 | 25 |
| MU39.1 | JBHHKY000000000 | 778 | 5,534,375 | 51.7 | 6299 | 55388 | 10081 | 26 | 100 |
| MU78.1 | JBHHKZ000000000 | 348 | 4,900,638 | 52.1 | 5159 | 276786 | 93293 | 5 | 16 |
| MU84.1 | JBHHLA000000000 | 321 | 5,024,192 | 51.8 | 5283 | 427770 | 93737 | 5 | 13 |
