# Supplementary material for: Genetic insights into antimicrobial resistance and virulence characteristics of Salmonella enterica isolated from Nile tilapia sourced from retail markets in Thailand
Source: BMC Microbiol. 2025 Nov 25;25:777. doi: 10.1186/s12866-025-04451-0 (PMC12649085; doi:10.1186/s12866-025-04451-0)
Supplement: Supplementary file 3 — Additional file 3: Table S3. Antibiogram and detailed genomic profile of Salmonella isolates from Nile Tilapia.Description of data: This file provides the antimicrobial susceptibility profiles and detailed genomic characteristics of Salmonella enterica isolates obtained from Nile Tilapia. [file 12866_2025_4451_MOESM3_ESM.docx]

**Additional file**

**Table S3**. Antibiogram and detailed genomic profile of *Salmonella* isolates from Nile Tilapia

| **Isolate ID** | **Sample type** | ***Salmonella* Serotype** | **Sequence type** | **Resistance pattern** | **Resistance gene** | **Efflux pump system gene** |
| --- | --- | --- | --- | --- | --- | --- |
| G26.3 | Gill | Stanley | ST34 | AMP-CHL-GEN-OXO-SUL-TRI-CPD-CTX-CAZ | *aac(6')-Iaa*, *sul3*, *cmlA1*, *aadA2*, *aph(3'')-Ib*, *aph(6)-Id*, *aadA2*, *dfrA12*, *_bla_*_CTX-M-55_, *qnrS1*, *aac(3)-IId* | *mdsA*, *mdsB*, *mdsC*, *mdtK* |
| G28.1 | Gill | Stanley | ST34 | AMP-CHL-GEN-OXO-SUL- TRI-CPD-CTX-CAZ | *ac(6')-Iaa*, *sul3*, *cmlA1*, *aadA2*, *aph(3'')-Ib*, *aph(6)-Id*, *aadA2*, *dfrA12*, *bla*_CTX-M-55_, *qnrS1*, *aac(3)-Iid* | *mdsA*, *mdsB*, *mdsC*, *mdtK* |
| G71.1 | Gill | Tallahassee | ST1541 | AMP-OXO-OTC-SUL-TET | *sul3*, *fosA7*, bla_TEM-1B_, *sul2*, *aph(3'')-Ib*, *aph(6)-Id*, *tet*(A), *qnrS1* | *mdsA*, *mdsB*, *mdsC*, *mdtK* K |
| G75.1 | Gill | Tallahassee | ST1541 | OXO-OTC-SUL-TET | *aac(6')-Iaa*, *sul2, aph(3'')-Ib*, *aph(6)-Id*, *tet*(A), *qnrS1* | *mdsA*, *mdsB*, *mdsC*, *mdtK* |
| G76.3 | Gill | Mbandaka | ST413 | OTC-STR-SUL-TET | *aac(6')-Iaa*, *sul2*, *aph(3'')-Ib*, *aph(6)-Id*, *tet*(A), *qnrS1* | *mdsA*, *mdsB*, *mdsC*, *mdtK* |
| I19.3 | Intestine | Othmarschen | ST469 | AMP-OTC-STR-TET | *aac(6')-Iaa*, *bla*_TEM-1B_, *aph(3'')-Ib*, *aph(6)-Id*, *tet*(A), *ant(3'')-Ia, lnu(F)* | *mdsA*, *mdsB*, *mdsC*, *mdtK* |
| I25.1 | Intestine | Senftenberg | ST2390 | AMP-CHL-GEN-OTC-SUL- TET-FLO-CPD-CTX | *aac(6')-Iaa*, *qnrS1*, *aac(3)-Iid*, *sul2*, *tet*(A), *floR*, *bla*_CTX-M-14_, *tet*(M) | *mdtK* |
| M51.1 | Meat | Escanaba | ST26 | AMP-OXO-OTC-SUL-TET | *aac(6')-Iaa* | *mdsA*, *mdsB*, *mdsC*, *mdtK* |
| M75.1 | Meat | Agona | ST13 | AMP-CHL-OXO-FLO | *aac(6')-Iaa*, *qnrS1*, *aac(3)-Iid*, *sul2*, *tet*(A), *floR, bla*_CTX-M-14_, *tet*(M)*, floR* | *mdsA*, *mdsB*, *mdsC*, *mdtK* |
| MU23.1 | Mucus | Brazil | ST446 | AMP-OXO-OTC-TET | *aac(6')-Iaa*, *qnrS1 bla*_TEM-1B_, *tet*(A), *mph*(A) | *mdsA*, *mdsB*, *mdsC*, *mdtK* |
| MU25.1 | Mucus | Stanley | ST34 | AMP-CHL-OXO-OTC-STR-SUL-TET-TRI | *aac(6')-Iaa*, *aadA2*, *qnrS1*, *bla*_TEM-1B_, *sul2*, *aph(3'')-Ib*, *aph(6)-Id*, *mph*(A), *bla_LAP-2_*, *tet*(B), *dfrA32*, *ere*(A), *sul1*, *catA2*, *aph(3')-Ia* | *mdsA*, *mdsB*, *mdsC*, *mdtK* |
| MU39.1 | Mucus | Agona | ST13 | AMP-OXO-OTC-STR-SUL-TET-TRI | *sul3*, *qnrS1*, *fosA7*, *bla*_TEM-1B_, *aph(3'')-Ib*, *aph(6)-Id*, *tet*(A), *dfrA14* | *mdsA*, *mdsB*, *mdsC*, *mdtK* |
| MU78.1 | Mucus | Mbandaka | ST413 | OXO-OTC-STR-SUL-TET | *qnrS1, sul2, aph(3'')-Ib,* *aph(6)-Id, tet*(A) | *mdsA*, *mdsB*, *mdsC*, *mdtK* |
| MU84.1 | Mucus | Newlands | ST321 | CHL-OXO-STR-SUL-TRI | *aac(6')-Iaa*, *sul3*, *cmlA1*, *aadA2*, *aph(3'')-Ib*, *aph(6)-Id* | *mdsA*, *mdsB*, *mdsC*, *mdtK* |
