# Supplementary material for: Genetic insights into antimicrobial resistance and virulence characteristics of Salmonella enterica isolated from Nile tilapia sourced from retail markets in Thailand
Source: BMC Microbiol. 2025 Nov 25;25:777. doi: 10.1186/s12866-025-04451-0 (PMC12649085; doi:10.1186/s12866-025-04451-0)
Supplement: Supplementary file 4 — Additional file 4: Table S4. Identified mobile genetic elements and their association with resistance genesDescription of data: This file presents a list of mobile genetic elements detected in S. enterica isolates and their associated AMR genes. [file 12866_2025_4451_MOESM4_ESM.docx]

**Additional file**

**Table S4**. Identified mobile genetic elements and their association with resistance genes.

| **Isolate ID** | **Contig ID** | **MGEs** | **Type** | **Size** | **Percent coverage** | **Percent identity** | **Number of substitutions** | **Associated resistance**  **gene** |
| --- | --- | --- | --- | --- | --- | --- | --- | --- |
| G26.3 | *S. enterica*_NODE_1 | MITEEc1 | Miniature inverted repeat | 123 | 100 | 93.5 | 8 |  |
|  | *S. enterica*_NODE_10 | MITEEc1 | Miniature inverted repeat | 123 | 100 | 93.5 | 8 |  |
|  | *S. enterica*_NODE_22 | ISKpn8 | Insertion sequence | 1443 | 100 | 94.46 | 80 |  |
|  | *S. enterica*_NODE_22 | IS102 | Insertion sequence | 1057 | 100 | 92.05 | 84 |  |
|  | *S. enterica*_NODE_37 | ISSen1 | Insertion sequence | 1312 | 100 | 99.62 | 5 |  |
|  | *S. enterica*_NODE_46 | ISEc9 | Insertion sequence | 1656 | 100 | 100 | 0 | *qnrS1*, *bla*_CTX-M-55_ |
|  | *S. enterica*_NODE_77 | IS6100 | Insertion sequence | 880 | 100 | 100 | 0 |  |
| G28.1 | *S. enterica*_NODE_1 | MITEEc1 | Miniature inverted repeat | 123 | 100 | 93.05 | 8 |  |
|  | *S. enterica*_NODE_10 | MITEEc1 | Miniature inverted repeat | 123 | 100 | 93.05 | 8 |  |
|  | *S. enterica*_NODE_37 | ISSen1 | Insertion sequence | 1312 | 100 | 99.62 | 5 |  |
|  | *S. enterica*_NODE_46 | ISEc9 | Insertion sequence | 1656 | 100 | 100 | 0 | *qnrS1*, *bla*_CTX-M-55_ |
|  | *S. enterica*_NODE_53 | IS5075 | Insertion sequence | 1327 | 100 | 93.22 | 90 |  |
|  | *S. enterica*_NODE_76 | IS6100 | Insertion sequence | 880 | 100 | 100 | 0 |  |
| G71.1 | *S. enterica*_NODE_23 | IS26 | Insertion sequence | 820 | 100 | 99.88 | 1 | *aph(6)-Id*, *tet*(A), *aph(3")-Ib*, *sul2* |
|  | *S. enterica*_NODE_2 | MITEEc1 | Insertion sequence | 120 | 97.56 | 94.17 | 7 |  |
| G75.1 | *S. enterica*_NODE_5 | MITEEc1 | Miniature inverted repeat | 123 | 100 | 93.05 | 8 |  |
|  | *S. enterica*_NODE_2 | MITEEc1 | Insertion sequence | 120 | 97.56 | 94.17 | 7 |  |
|  | *S. enterica*_NODE_5 | MITEEc1 | Miniature inverted repeat | 123 | 100 | 93.05 | 8 |  |
| G76.3 | *S. enterica*_NODE_32 | IS26 | Insertion sequence | 820 | 100 | 99.88 |  | *aph(6)-Id*, *tet*(A), *aph(3")-Ib*, *sul2* |
|  | *S. enterica*_NODE_3 | ISEcl10 | Insertion sequence | 1207 | 100 | 95.03 | 60 |  |
| I19.3 | *S. enterica*_NODE_1 | MITEEc1 | Miniature inverted repeat | 123 | 100 | 93.5 | 8 |  |
|  | *S. enterica*_NODE_8 | MITEEc1 | Miniature inverted repeat | 121 | 98.37 | 95.04 | 6 |  |
|  | *S. enterica*_NODE_19 | ISEcl10 | Insertion sequence | 1191 | 98.67 | 93.04 | 68 |  |
|  | *S. enterica*_NODE_22 | ISEcl10 | Insertion sequence | 1207 | 100 | 95.11 | 59 |  |
|  | *S. enterica*_NODE_56 | ISKpn2 | Insertion sequence | 1306 | 99.77 | 97.17 | 34 |  |
|  | *S. enterica*_NODE_116 | ISEhe3 | Insertion sequence | 1229 | 99.84 | 94.80% | 62 |  |
|  | *S. enterica*_NODE_125 | ISKpn26 | Insertion sequence | 1196 | 100 | 99.50% | 6 |  |
|  | *S. enterica*_NODE_195 | IS26 | Insertion sequence | 820 | 100 | 100 | 0 |  |
| I25.1 | *S. enterica*_NODE_67 | ISVsa3 | Insertion sequence | 977 | 100 | 100 | 0 | *sul2*, *floR* |
|  | *S. enterica*_NODE_77 | ISEc9 | Insertion sequence | 1656 | 100 | 99.88 | 2 | *bla*_CTX-M-14_, *qnrS1* |
|  | *S. enterica*_NODE_77 | IS102 | Insertion sequence | 1057 | 100 | 92.62 | 78 |  |
|  | *S. enterica*_NODE_1 | ISKpn2 | Insertion sequence | 1296 | 98.93 | 97 | 31 |  |
|  | *S. enterica*_NODE_8 | MITEEc1 | Miniature inverted repeat | 123 | 100 | 94.31 | 7 |  |
|  | *S. enterica*_NODE_15 | ISSen6 | Insertion sequence | 1818 | 100 | 98.51 | 27 |  |
|  | *S. enterica*_NODE_18 | MITEEc1 | Miniature inverted repeat | 123 | 100 | 92.68 | 9 |  |
|  | *S. enterica*_NODE_30 | ISSso4 | Insertion sequence | 2638 | 97.76 | 96.28 | 95 |  |
|  | *S. enterica*_NODE_94 | ISSen1 | Insertion sequence | 1312 | 100 | 99.16 | 11 |  |
|  | *S. enterica*_NODE_94 | ISEcl10 | Insertion sequence | 1206 | 99.92 | 94.28 | 68 |  |
|  | *S. enterica*_NODE_97 | ISEhe3 | Insertion sequence | 1229 | 99.84 | 94.72 | 63 |  |
|  | *S. enterica*_NODE_158 | ISEcl10 | Insertion sequence | 1207 | 100 | 93.54 | 78 |  |
| M51.1 | *S. enterica*_NODE_2 | MITEEc1 | Miniature inverted repeat | 123 | 100 | 93.5 | 8 |  |
|  | *S. enterica*_NODE_6 | ISSty2 | Insertion sequence | 1259 | 100 | 98.25 | 22 |  |
|  | *S. enterica*_NODE_8 | MITEEc1 | Miniature inverted repeat | 123 | 100 | 93.5 | 8 |  |
| M75.1 | *S. enterica*_NODE_1 | IS26 | Insertion sequence | 820 | 100 | 99.63 | 3 | *aac(6')-Iaa*, *floR* |
|  | *S. enterica*_NODE_2 | MITEEc1 | Miniature inverted repeat | 123 | 100 | 94.31 | 7 |  |
|  | *S. enterica*_NODE_3 | ISKpn19 | Insertion sequence | 2851 | 100 | 100 | 0 | *qnrS1* |
|  | *S. enterica*_NODE_5 | ISKpn2 | Insertion sequence | 1306 | 99.77 | 97.25 | 33 |  |
|  | *S. enterica*_NODE_9 | ISEcl10 | Insertion sequence | 1207 | 100 | 93.29 | 81 |  |
|  | *S. enterica*_NODE_10 | ISSen6 | Insertion sequence | 1818 | 100 | 98.9 | 20 |  |
|  | *S. enterica*_NODE_31 | ISSen1 | Insertion sequence | 1312 | 100 | 98.93 | 14 |  |
| MU23.1 | *S. enterica*_NODE_3 | ISKpn2 | Insertion sequence | 1306 | 99.85 | 97.48 | 31 |  |
|  | *S. enterica*_NODE_6 | ISEcl10 | Insertion sequence | 1194 | 98.92 | 93.12 | 70 |  |
|  | *S. enterica*_NODE_10 | MITEEc1 | Miniature inverted repeat | 123 | 100 | 933.5 | 8 |  |
|  | *S. enterica*_NODE_12 | ISEc17 | Insertion sequence | 1258 | 100 | 99.76 | 3 |  |
|  | *S. enterica*_NODE_22 | IS6100 | Insertion sequence | 880 | 100 | 100 | 0 | *bla*_TEM-1B_, *tetA* |
|  | *S. enterica*_NODE_23 | ISKpn19 | Insertion sequence | 1251 | 100 | 100 | 0 | *mph*(A), *qnrS1* |
|  | *S. enterica*_NODE_23 | MITEEc1 | Miniature inverted repeat | 123 | 100 | 93.5 | 8 |  |
|  | *S. enterica*_NODE_33 | ISSen1 | Insertion sequence | 1312 | 100 | 99.54 | 6 |  |
|  | *S. enterica*_NODE_36 | IS26 | Insertion sequence | 820 | 100 | 100 | 0 |  |
| MU25.1 | *S. enterica*_NODE_1 | MITEEc1 | Miniature inverted repeat | 123 | 100 | 93.5 | 8 |  |
|  | *S. enterica*_NODE_11 | MITEEc1 | Miniature inverted repeat | 123 | 100 | 93.5 | 8 |  |
|  | *S. enterica*_NODE_12 | ISKpn19 | Insertion sequence | 2851 | 100 | 100 | 0 | *bla*_LAP-2_, *qnrS1, mph*(A) |
|  | *S. enterica*_NODE_34 | ISSen1 | Insertion sequence | 1312 | 100 | 99.62 | 5 |  |
|  | *S. enterica*_NODE_56 | IS100 | Insertion sequence | 1954 | 99.95 | 99.33 | 12 |  |
|  | *S. enterica*_NODE_68 | ISVsa5 | Insertion sequence | 1329 | 100 | 98.8 | 16 |  |
|  | *S. enterica*_NODE_75 | S102 | Insertion sequence | 1057 | 100 | 92.9 | 75 |  |
| MU39.1 | *S. enterica*_NODE_5 | ISSen6 | Insertion sequence | 1818 | 100 | 98.95 | 19 |  |
|  | *S. enterica*_NODE_13 | MITEEc1 | Miniature inverted repeat | 123 | 100 | 93.5 | 8 |  |
|  | *S. enterica*_NODE_31 | ISKpn2 | Insertion sequence | 1306 | 99.77 | 97.25 | 33 |  |
|  | *S. enterica*_NODE_52 | ISKpn50 | Insertion sequence | 2433 | 99.1 | 93.52 | 137 |  |
|  | *S. enterica*_NODE_80 | ISEcl10 | Insertion sequence | 1207 | 100 | 93.87 | 74 |  |
|  | *S. enterica*_NODE_83 | ISKpn19 | Insertion sequence | 2851 | 100 | 100 | 0 | *tet(*A), *qnrS1* |
|  | *S. enterica*_NODE_117 | IS903 | Insertion sequence | 1057 | 100 | 94.89 | 54 | *aph(3")-Ib*, *aph(6)-Id* |
|  | *S. enterica*_NODE_218 | ISSen1 | Insertion sequence | 1312 | 100 | 98.93 | 14 |  |
|  | *S. enterica*_NODE_272 | IS26 | Insertion sequence | 820 | 100 | 100 | 0 |  |
| MU78.1 | *S. enterica*_NODE_2 | ISEcl10 | Insertion sequence | 1207 | 100 | 95.03 | 60 |  |
|  | *S. enterica*_NODE_3 | ISEcl10 | Insertion sequence | 1207 | 100 | 93.79 | 75 |  |
|  | *S. enterica*_NODE_3 | IS630 | Insertion sequence | 1152 | 99.91 | 90.89 | 104 |  |
|  | *S. enterica*_NODE_13 | ISKpn2 | Insertion sequence | 1305 | 99.62 | 97.02 | 34 |  |
|  | *S. enterica*_NODE_14 | MITEEc1 | Miniature inverted repeat | 123 | 100 | 93.5 | 8 |  |
|  | *S. enterica*_NODE_25 | IS26 | Insertion sequence | 820 | 100 | 99.88 | 1 | *aph(3'')-Ib*, *aph(6)-Id*, *qnrS1*, *sul2*, *tet*(A) |
| MU84.1 | *S. enterica*_NODE_1 | MITEEc1 | Insertion sequence | 123 | 100 | 94.31 | 7 |  |
|  | *S. enterica*_NODE_9 | ISKpn18 | Insertion sequence | 1283 | 98.47 | 100 | 0 | *aph(3'')-Ib*, *aph(6)-Id* |
|  | *S. enterica*_NODE_9 | IS421 | Insertion sequence | 1339 | 99.78 | 99.78 | 0 |  |
|  | *S. enterica*_NODE_9 | Tn6024 | Unit transposon | 32409 | 99.98 | 100 | 12 |  |
|  | *S. enterica*_NODE_29 | ISSen1 | Insertion sequence | 1312 | 100 | 99.09 | 12 |  |
