# Supplementary material for: Genetic insights into antimicrobial resistance and virulence characteristics of Salmonella enterica isolated from Nile tilapia sourced from retail markets in Thailand
Source: BMC Microbiol. 2025 Nov 25;25:777. doi: 10.1186/s12866-025-04451-0 (PMC12649085; doi:10.1186/s12866-025-04451-0)
Supplement: Supplementary file 5 — Additional file 5: Table S5. Distribution variability of virulence genes across Salmonella enterica isolates.Description of data: This file shows the variability in virulence gene profiles across all S. enterica isolates examined in the study. [file 12866_2025_4451_MOESM5_ESM.docx]

**Additional file**

**Table S5.** Distribution variability of virulence genes across *Salmonella enterica* isolates.

| **Virulence genes** | **Virulence factors** | **Present** | **Absent** | **Remarks** |
| --- | --- | --- | --- | --- |
| *avrA* | SP-1 T3SS | 14 |  | All isolates |
| *cdtB* | cytolethal distending toxin | 1 | 13 | MU84.1 |
| *csgA* | Curli | 14 |  | All isolates |
| *csgB* | Curli | 14 |  | All isolates |
| *csgC* | Curli | 14 |  | All isolates |
| *csgD* | Curli | 14 |  | All isolates |
| *csgE* | Curli | 14 |  | All isolates |
| *csgF* | Curli | 14 |  | All isolates |
| *csgG* | Curli | 14 |  | All isolates |
| *entA* | Enterobactin | 11 | 3 | Absent in G71.1, MU23.1, and MU84.1 |
| *entB* | Enterobactin | 13 | 1 | Absent in G71.1 |
| *faeC* | K88 pilli/F4 fimbriae | 7 | 7 | G71.1, G76.1, I25.1, and M23.1, MU39.1, MU78.1, and MU84.1 |
| *faeD* | K88 pilli/F4 fimbriae | 6 | 8 | G71.1, G76.1, M23.1, and MU39.1, MU78.1, and MU84.1 |
| *faeE* | K88 pilli/F4 fimbriae | 7 | 7 | G71.1, G76.1, I25.1, and M23.1, MU39.1, and MU78.1, MU84.1 |
| *fepC* | Ferroenterobactin | 14 | 0 | All isolates |
| *fepG* | Ferroenterobactin | 14 | 0 | All isolates |
| *fimC* | Type 1 fimbriae | 14 | 0 | All isolates |
| *fimD* | Type 1 fimbriae | 13 | 1 | Absent in MU39.1 |
| *fimF* | Type 1 fimbriae | 14 |  | All isolates |
| *fimH* | Type 1 fimbriae | 14 |  | All isolates |
| *fimI* | Type 1 fimbriae | 14 |  | All isolates |
| *gogB* | SP-2 T3SS | 5 | 9 | G26.3, G28.1, M51.1, and MU23.1 and MU25.1 |
| *invA* | SPI-1 T3SS | 14 |  | All isolates |
| *invB* | SPI-1 T3SS | 14 |  | All isolates |
| *invC* | SPI-1 T3SS | 14 |  | All isolates |
| *invE* | SPI-1 T3SS | 14 |  | All isolates |
| *invF* | SPI-1 T3SS | 14 |  | All isolates |
| *invG* | SPI-1 T3SS | 14 |  | All isolates |
| *invH* | SPI-1 T3SS | 14 |  | All isolates |
| *invI* | SPI-1 T3SS | 14 |  | All isolates |
| *invJ* | SPI-1 T3SS | 14 |  | All isolates |
| *lpfA* | Long polar fimbriae | 12 | 2 | Absent in G75.1, MU84.1 |
| *lpfB* | Long polar fimbriae | 12 | 2 | Absent in G75.1, MU84.1 |
| *lpfC* | Long polar fimbriae | 12 | 2 | Absent in G75.1, MU84.1 |
| *lpfD* | Long polar fimbriae | 6 | 8 | G26.3, G28.1, G71.1, and M51.1, MU23.1, and MU25.1 |
| *lpfE* | Long polar fimbriae | 12 | 2 | Absent in G75.1, MU84.1 |
| *mgtB* | Magnesium transport protein | 14 |  | All isolates |
| *mgtC* | Magnesium transport protein | 14 |  | All isolates |
| *mig-14* | Antimicrobial resistance peptide | 14 |  | All isolates |
| *misL* | Putative autotransporter | 14 |  | All isolates |
| *orgA* | SPI-1 T3SS | 14 |  | All isolates |
| *orgB* | SPI-1 T3SS | 14 |  | All isolates |
| *orgC* | SPI-1 T3SS | 14 |  | All isolates |
| *pipB* | SP-2 T3SS | 14 |  | All isolates |
| *pipB2* | SP-2 T3SS | 13 | 1 | MU84.1 |
| *prgH* | SPI-1 T3SS | 14 |  | All isolates |
| *prgI* | SPI-1 T3SS | 14 |  | All isolates |
| *prgJ* | SPI-1 T3SS | 14 |  | All isolates |
| *prgK* | SPI-1 T3SS | 14 |  | All isolates |
| *shdA* | autotransporter-like protein | 2 | 12 | M51.1 and MU23.1 |
| *sicA* | SPI-1 T3SS | 14 |  | All isolates |
| *sicP* | SPI-1 T3SS | 14 |  | All isolates |
| *sifA* | SPI-2 T3SS | 14 |  | All isolates |
| *sifB* | SPI-2 T3SS | 14 |  | All isolates |
| *sinH* | sinH initimin-like protein | 13 | 1 | Absent in G71.1 |
| *sipA/sspA* | SPI-1 T3SS | 13 | 1 | Absent in G71.1 |
| *sipB/sspB* | SPI-1 T3SS | 14 |  | All isolates |
| *sipC/sspC* | SPI-1 T3SS | 14 |  | All isolates |
| *sipD* | SPI-1 T3SS | 14 |  | All isolates |
| *slrP* | SPI-2 T3SS | 13 | 1 | Absent in G71.1 |
| *sodCI* | SPI-2 T3SS | 3 | 11 | G26.3, G28.1, and MU25.1 |
| *sopA* | SPI-1 T3SS | 14 |  | All isolates |
| *sopB/sigD* | SPI-1 T3SS | 14 |  | All isolates |
| *sopD* | SPI-1 T3SS | 14 |  | All isolates |
| *sopD2* | SPI-1 T3SS | 13 | 1 | Absent in G71.1 |
| *sopE2* | SPI-1 T3SS | 12 | 2 | Absent in G71.1 and MU39.1 |
| *spaO* | SPI-1 T3SS | 14 |  | All isolates |
| *spaP* | SPI-1 T3SS | 14 |  | All isolates |
| *spaQ* | SPI-1 T3SS | 14 |  | All isolates |
| *spaR* | SPI-1 T3SS | 14 |  | All isolates |
| *spaS* | SPI-1 T3SS | 14 |  | All isolates |
| *spiC/ssaB* | SPI-2 T3SS | 14 |  | All isolates |
| *sptP* | SPI-1 T3SS | 14 |  | All isolates |
| *ssaC* | SPI-2 T3SS | 14 |  | All isolates |
| *ssaD* | SPI-2 T3SS | 14 |  | All isolates |
| *ssaE* | SPI-2 T3SS | 14 |  | All isolates |
| *ssaG* | SPI-2 T3SS | 14 |  | All isolates |
| *ssaH* | SPI-2 T3SS | 14 |  | All isolates |
| *ssaI* | SPI-2 T3SS | 14 |  | All isolates |
| *ssaJ* | SPI-2 T3SS | 14 |  | All isolates |
| *ssaK* | SPI-2 T3SS | 14 |  | All isolates |
| *ssaL* | SPI-2 T3SS | 14 |  | All isolates |
| *ssaM* | SPI-2 T3SS | 14 |  | All isolates |
| *ssaN* | SPI-2 T3SS | 14 |  | All isolates |
| *ssaO* | SPI-2 T3SS | 14 |  | All isolates |
| *ssaP* | SPI-2 T3SS | 14 |  | All isolates |
| *ssaQ* | SPI-2 T3SS | 14 |  | All isolates |
| *ssaR* | SPI-2 T3SS | 14 |  | All isolates |
| *ssaS* | SPI-2 T3SS | 14 |  | All isolates |
| *ssaT* | SPI-2 T3SS | 14 |  | All isolates |
| *ssaU* | SPI-2 T3SS | 14 |  | All isolates |
| *ssaV* | SPI-2 T3SS | 14 |  | All isolates |
| *sscA* | SPI-2 T3SS | 14 |  | All isolates |
| *sscB* | SPI-2 T3SS | 14 |  | All isolates |
| *sseA* | SPI-2 T3SS | 14 |  | All isolates |
| *sseB* | SPI-2 T3SS | 13 |  | Absent in G71.1 |
| *sseC* | SPI-2 T3SS | 14 |  | All isolates |
| *sseD* | SPI-2 T3SS | 14 |  | All isolates |
| *sseE* | SPI-2 T3SS | 14 |  | All isolates |
| *sseF* | SPI-2 T3SS | 14 |  | All isolates |
| *sseG* | SPI-2 T3SS | 14 |  | All isolates |
| *sseI/srfH* | SPI-2 T3SS | 3 | 11 | G26.3, G28.1, and MU25.1 |
| *sseJ* | SPI-2 T3SS | 13 | 1 | Absent in G71.1 |
| *sseK1* | SPI-2 T3SS | 13 | 1 | Absent in M51.1 |
| *sseK2* | SPI-2 T3SS | 10 | 4 | Absent in M51.1 and M75.1, MU23.1 and MU84.1 |
| *sseL* | SPI-2 T3SS | 13 | 1 | Absent in MU39.1 |
| *sspH1* | SPI-2 T3SS | 2 | 12 | G71.1 and I25.1 |
| *sspH2* | SPI-2 T3SS | 6 | 8 | G26.3, G28.1, G75.1, M51.1, MU23.1, and MU25.1 |
| *steA* | SPI-2 T3SS | 13 | 1 | Absent in G71.1 |
| *steB* | SPI-2 T3SS | 13 | 1 | Absent in G71.1 |
| *steC* | SPI-2 T3SS | 12 | 2 | Absent in G71.1 and MU39.1 |
| *tcpC* | Tir containing protein | 1 | 13 | I25.1 |
