# Supplementary material for: Genetic insights into antimicrobial resistance and virulence characteristics of Salmonella enterica isolated from Nile tilapia sourced from retail markets in Thailand
Source: BMC Microbiol. 2025 Nov 25;25:777. doi: 10.1186/s12866-025-04451-0 (PMC12649085; doi:10.1186/s12866-025-04451-0)
Supplement: Supplementary file 6 — Addtional file 6: Figure S1. Maximum likelihood phylogenetic tree of Salmonella enterica isolates from Nile tilapia in Thailand and reference genomes from GenBank. A phylogenetic tree was constructed using the maximum likelihood method based on core genome alignments of Salmonella enterica isolates sequenced in this study (n = 14), along with representative reference genomes obtained from GenBank. Isolates are labeled by genus and serovar, followed by accession numbers for GenBank references. Study isolates are denoted by alphabetical codes with numerical identifiers. Colors indicate the source of isolation, including fish tissues (gill, mucus, intestine, meat), humans, poultry, wastewater, food, farms, and wet markets. Country labels denote the geographical origin of each isolate. The scale bar (0.01) represents the number of nucleotide substitutions per site. [file 12866_2025_4451_MOESM6_ESM.docx]

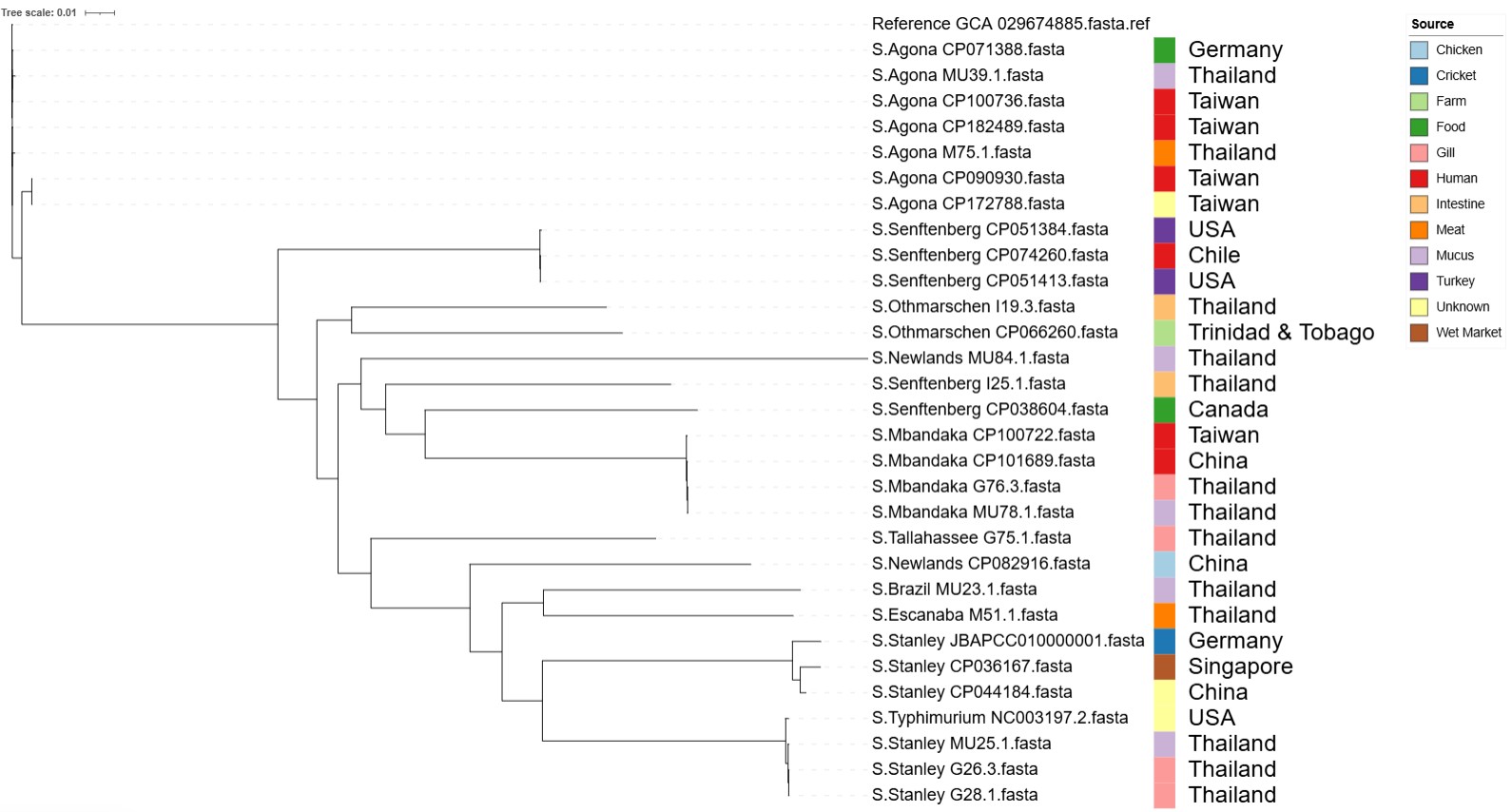


**Additional file**

**Figure S1**. Maximum likelihood phylogenetic tree of *Salmonella enterica* isolates from Nile tilapia in Thailand and reference genomes from GenBank. A phylogenetic tree was constructed using the maximum likelihood method based on core genome alignments of *Salmonella enterica* isolates sequenced in this study (n = 14), along with representative reference genomes obtained from GenBank. Isolates are labeled by genus and serovar, followed by accession numbers for GenBank references.

Note: Study isolates are denoted by alphabetical codes with numerical identifiers. Colors indicate the source of isolation, including fish tissues (gill, mucus, intestine, meat), humans, poultry, wastewater, food, farms, and wet markets. Country labels denote the geographical origin of each isolate. The scale bar (0.01) represents the number of nucleotide substitutions per site.
