# Supplementary material for: Genetic insights into antimicrobial resistance and virulence characteristics of Salmonella enterica isolated from Nile tilapia sourced from retail markets in Thailand
Source: BMC Microbiol. 2025 Nov 25;25:777. doi: 10.1186/s12866-025-04451-0 (PMC12649085; doi:10.1186/s12866-025-04451-0)
Supplement: Supplementary file 7 [file 12866_2025_4451_MOESM1_ESM.docx]

Additional file 

Table S1. Minimum inhibitory concentration, zone of inhibition and resistance gene profile of multidrug resistant isolates. 

| Isolate ID | Resistance pattern | Resistance gene | AMP | CHL | CIP | ENR | GEN | OXO | OTC | STR | SUL | TET | TRI | FLOR | CPD | CTX | CAZ |
| --- | --- | --- | --- | --- | --- | --- | --- | --- | --- | --- | --- | --- | --- | --- | --- | --- | --- |
| G26.3 | AMP-CHL-GEN-OXO-SUL-TRI-CPD-CTX-CAZ | aac(6')-Iaa, sul3, cmlA1, aadA2, aph(3'')-Ib, aph(6)-Id, aadA2, dfrA12, blaCTX-M-55, qnrS1, aac(3)-IId | 2048 | 64 | 0.5 | 1 | 32 | 2 | 2 | 16 | 4096 | 1 | 512 | 24 | 0 | 0 | 12 |
| G28.1 | AMP-CHL-GEN-OXO-SUL-TRI-CPD-CTX-CAZ | ac(6')-Iaa, sul3, cmlA1, aadA2, aph(3'')-Ib, aph(6)-Id, aadA2, dfrA12, blaCTX-M-55, qnrS1, aac(3)-Iid | 2048 | 64 | 0.5 | 1 | 32 | 2 | 2 | 4 | 4096 | 1 | 512 | 24 | 0 | 0 | 13 |
| G71.1 | AMP-OXO-OTC-SUL-TET | sul3, fosA7, blaTEM-1B, sul2, aph(3'')-Ib, aph(6)-Id, tet(A), qnrS1 | 1024 | 4 | 0.5 | 1 | 0.5 | 2 | 256 | 8 | 4096 | 32 | 0.125 | 29 | 30 | 35 | 31 |
| G75.1 | OXO-OTC-SUL-TET | aac(6')-Iaa, sul2, aph(3'')-Ib, aph(6)-Id, tet(A), qnrS1 | 8 | 2 | 0.5 | 1 | 0.25 | 4 | 512 | 8 | 4096 | 64 | 0.125 | 30 | 34 | 36 | 31 |
| G76.3 | OTC-STR-SUL-TET | aac(6')-Iaa, sul2, aph(3'')-Ib, aph(6)-Id, tet(A), qnrS1 | 4 | 4 | 0.5 | 1 | 1 | 1 | 512 | 32 | 4096 | 64 | 0.125 | 28 | 32 | 34 | 32 |
| I19.3 | AMP-OTC-STR-TET | aac(6')-Iaa, blaTEM-1B, aph(3'')-Ib, aph(6)-Id, tet(A), ant(3'')-Ia, lnu(F) | 2048 | 8 | 0.03 | 0.06 | 0.5 | 0.5 | 4 | 512 | 32 | 32 | 0.25 | 8 | 28 | 32 | 28 |
| I25.1 | AMP-CHL-GEN-OTC-SUL-TET-FLO-CPD-CTX | aac(6')-Iaa, qnrS1, aac(3)-Iid, sul2, tet(A), floR, blaCTX-M-14, tet(M) | 2048 | 256 | 0.25 | 1 | 64 | 1 | 256 | 4 | 4096 | 128 | 0.5 | 16 | 0 | 5 | 23 |
| M51.1 | AMP-OXO-OTC-SUL | aac(6')-Iaa | 32 | 8 | 0.06 | 0.25 | 1 | 2 | 16 | 4 | 32 | 4 | 0.5 | 27 | 28 | 32 | 23 |
| M75.1 | AMP-CHL-OXO-FLO | aac(6')-Iaa, qnrS1, aac(3)-Iid, sul2, tet(A), floR, blaCTX-M-14, tet(M), floR | 32 | 128 | 1 | 2 | 0.5 | 4 | 8 | 4 | 16 | 0.5 | 0.25 | 16 | 30 | 35 | 24 |
| MU23.1 | AMP-OXO-OTC-TET | aac(6')-Iaa, qnrS1 blaTEM-1B, tet(A), mph(A) | 1024 | 4 | 0.5 | 1 | 1 | 4 | 256 | 4 | 16 | 64 | 0.25 | 25 | 29 | 30 | 23 |
| MU25.1 | AMP-CHL-OXO-OTC-STR-SUL-TET-TRI | aac(6')-Iaa, aadA2, qnrS1, blaTEM-1B, sul2, aph(3'')-Ib, aph(6)-Id, mph(A), blaLAP-2, tet(B), dfrA32, ere(A), sul1, catA2, aph(3')-Ia | 2048 | 512 | 0.5 | 1 | 1 | 2 | 512 | 512 | 4096 | 128 | 512 | 29 | 31 | 34 | 24 |
| MU39.1 | AMP-OXO-OTC-STR-SUL-TET-TRI | sul3, qnrS1, fosA7, blaTEM-1B, aph(3'')-Ib, aph(6)-Id, tet(A), dfrA14 | 1024 | 8 | 0.5 | 1 | 1 | 4 | 512 | 256 | 4096 | 64 | 512 | 29 | 29 | 31 | 23 |
| MU78.1 | OXO-OTC-STR-SUL-TET | qnrS1, sul2, aph(3'')-Ib, aph(6)-Id, tet(A) | 8 | 4 | 0.5 | 1 | 0.5 | 4 | 512 | 32 | 4096 | 64 | 0.25 | 31 | 33 | 33 | 26 |
| MU84.1 | CHL-OXO-STR-SUL-TRI | aac(6')-Iaa, sul3, cmlA1, aadA2, aph(3'')-Ib, aph(6)-Id | 8 | 128 | 1 | 2 | 1 | 4 | 8 | 64 | 4096 | 2 | 512 | 27 | 30 | 34 | 23 |

Portion shaded yellow indicates zone of inhibition values.
